# Ventricular anatomical complexity and sex differences impact predictions from electrophysiological computational models

**DOI:** 10.1371/journal.pone.0263639

**Published:** 2023-02-13

**Authors:** Pablo Gonzalez-Martin, Federica Sacco, Constantine Butakoff, Ruben Doste, Carlos Bederian, Lilian K. Gutierrez Espinosa de los Monteros, Guillaume Houzeaux, Paul A. Iaizzo, Tinen L. Iles, Mariano Vazquez, Jazmin Aguado-Sierra

**Affiliations:** 1 Barcelona Supercomputing Center, Barcelona, Spain; 2 ELEM Biotech S.L., Barcelona, Spain; 3 Physense, Department of Information and Communication Technologies, Universitat Pompeu Fabra, Barcelona, Spain; 4 Department of Computer Science, University of Oxford, Oxford, United Kingdom; 5 Instituto de Física Enrique Gaviola - CONICET, Córdoba, Argentina; 6 Centro Nacional de Investigaciones Cardiovasculares Carlos III, Madrid, Spain; 7 Visible Heart^®^ Laboratories, Department of Surgery and the Institute for Engineering in Medicine, University of Minnesota, Minneapolis, MN, United States of America; 8 University of Minnesota Medical School, Minneapolis, MN, United States of America; Universidad de Zaragoza, SPAIN

## Abstract

The aim of this work was to analyze the influence of sex hormones and anatomical details (trabeculations and false tendons) on the electrophysiology of healthy human hearts. Additionally, sex- and anatomy-dependent effects of ventricular tachycardia (VT) inducibility are presented. To this end, four anatomically normal, human, biventricular geometries (two male, two female), with identifiable trabeculations, were obtained from high-resolution, ex-vivo MRI and represented by detailed and smoothed geometrical models (with and without the trabeculations). Additionally one model was augmented by a scar. The electrophysiology finite element model (FEM) simulations were carried out, using O’Hara-Rudy human myocyte model with sex phenotypes of Yang and Clancy. A systematic comparison between detailed vs smooth anatomies, male vs female normal hearts was carried out. The heart with a myocardial infarction was subjected to a programmed stimulus protocol to identify the effects of sex and anatomical detail on ventricular tachycardia inducibility. All female hearts presented QT-interval prolongation however the prolongation interval in comparison to the male phenotypes was anatomy-dependent and was not correlated to the size of the heart. Detailed geometries showed QRS fractionation and increased T-wave magnitude in comparison to the corresponding smoothed geometries. A variety of sustained VTs were obtained in the detailed and smoothed male geometries at different pacing locations, which provide evidence of the geometry-dependent differences regarding the prediction of the locations of reentry channels. In the female phenotype, sustained VTs were induced in both detailed and smooth geometries with RV apex pacing, however no consistent reentry channels were identified. Anatomical and physiological cardiac features play an important role defining risk in cardiac disease. These are often excluded from cardiac electrophysiology simulations. The assumption that the cardiac endocardium is smooth may produce inaccurate predictions towards the location of reentry channels in in-silico tachycardia inducibility studies.

## Introduction

Imaging-based cardiac computational simulations have opened a new paradigm for personalized medicine applied to complex structural pathologies in search for a better understanding and stratification of disease. Computational tools are also being recognized as key contributors to the reduction of the time and economical burden of regulatory pathways for both the pharmaceutical and the medical device industry aiming towards a more efficient design and development of drugs, treatments, and ultimately to achieve better outcomes in the clinic.

Cardiac electrophysiology modelling has been a primordial target for computer simulations. Seventy years of research on mathematical modelling of cardiac electrophysiology has produced a wealth of information regarding single cell dynamics that led to various mathematical models describing the ventricular function of the human heart [1]. However, even when detailed mathematical cell models are available, the geometry of the ventricle, used for the simulations is often simplified. The simplifications are dictated by the imaging modality available for the model construction and computational power available. The most typical imaging modality used for model construction is Magnetic Resonance Imaging (MRI), acquired in slices, with, typically, good within-slice resolution but 5–10mm distance between slices. This low spatial resolution is insufficient for capturing the complexity of human endocardium (i.e., trabeculae and false tendons). It is therefore important to quantify the potential errors produced by the geometrical simplifications, given the imaging data obtained in the every-day clinic, as the endocardial structures may create electrical bridges and may play a crucial role in efficient action potential propagation.

Functional sex differences is another aspect of the computational cardiac models that is generally ignored. Understanding the sex-specific cardiac pathophysiology is still in its infancy. These differences may play an important role in arrhythmogenesis [2]. For example, Yang et al. [3], reported genomic-based differences within ion channel expressions, leading to longer cardiac action potential durations (APDs) in females than in males. Female human hearts have been shown to elicit lower expressions of the genes responsible for cardiac repolarizing potassium currents, and Connexin43. Considering the differences in ion channel sub-unit expressions is important when studying sex-specific physio-pathologies, in particular sex-specific arrhythmic risks.

All human cardiac ventricles have a sponge-like architecture inside the cavity, different in every heart [4]. False tendons establish fast conducting shortcuts within the ventricular cavities, but their small diameter makes them impossible to reproduce on patient-specific cardiac computational models using conventional clinical imaging tools. Nevertheless, they play a role in the overall cardiac pathophysiology.

To date, there have been a few computational models studying the influences of the position and amount of both trabeculae and False Tendons (FTs) FTs on the overall cardiac electrophysiology (Bishop et al. [5], Bordas et al. [6], Lange et al. [7], Connolly et al. [8] and Galappaththinge et al. [9]).

Firstly, in 2010, [5] developed a detailed rabbit biventricular heart model from high-resolution magnetic resonance images (MRI), characterized by some trabeculae but devoid of any long FTs. In their work, they compared the activation patterns generated by an anatomically detailed model with a smoothed equivalent. Interestingly, they reported that the presence of trabeculae provided a short-cut pathway for the excitation, leading to regional differences in the activation between the two models. Even though this work can be considered as first insights to the functions and influence of the presence of trabeculae on the whole cardiac activation, their study had several important limitations. First, the activation was initiated with a single stimulus at the ventricular apex; which is far from a physiological cardiac activation. Second, no FTs were reconstructed and the geometry employed was a rabbit biventricular anatomy, which cannot be directly compared to the human heart.

Subsequently, the work of [5] was further improved by Bordas and co-workers [6], who incorporated a detailed description of the free-running part of the specialized conduction system (SCS) into a detailed rabbit heart model. Their simulations were carried out with both a full SCS model and a simplified model (without the free-running Purkinje system, FRPS). They observed that the inclusion of the FRPS resulted in slightly faster and more coordinated activations of the ventricles compared to imposed smoothed geometries. The FTs influence on cardiac electrophysiology was further analyzed by [7], who reconstructed a smooth left ventricular (LV) human heart model from CT images, characterized only by the two papillary muscles (PMs). In this study, the authors computationally generated a Purkinje system and varied the relative positions of the main FTs in order to study how this would affect overall QRS durations. The same authors also simulated a left bundle branch block (LBBB) case and analyzed the roles of the FTs in this pathological scenario. They reported that the configurations with the FTs connecting the septum to the ventricular walls, resulted in the shortest mean QRS durations. Moreover, they suggested that the presence of FTs in the LBBB case was beneficial for improving overall cardiac function. The main limitations to their work, were the omissions of other anatomical details; e.g., the two LV PMs and RV anatomies were not included. More recently, [8] investigated the response of human heart trabeculations to low-energy monophasic shocks. They reported how induced shocks, generated local regions of depolarization on the distal side of trabeculations, became entirely detached from the endocardial surfaces, relative to the electrode position. Nevertheless, they noted that a ventricular tachycardia was induced using a prescribed stimulus, following an apically paced beat: this suggested how anodal shocks applied to episodes of sustained arrhythmia induced more small, isolated propagating wavefronts.

In related work, Galappaththige et al. [9] studied the roles of endocardial sub-structures on ventricular fibrillation (VF) dynamics: which are known to be caused by unstable reentrant electrical waves which rotate around such filaments. To do so, they utilized both detailed and smooth-walled biventricular 3D high-resolution rabbit heart geometries and studied VF dynamics with both anisotropic and isotropic conductivities. Interestingly, they observed that for the hearts with the anatomically detailed geometries, VF simulation results were similar to the complex dynamics observed experimentally. Whereas for the employed smoothed geometries, the number of filaments were reduced compared to the detailed heart. Moreover, they reported that endocardial sub-structures and anisotropies, both destabilized reentrant waves. Endocardial structures increased the number of filaments. In the smoothed cardiac models, anisotropy destabilized the VF dynamics, although the amounts of filaments were less as compared to those with the detailed cardiac geometries. None of these previous works explore the effect of detailed endocardial structures on ventricular tachycardia simulations and the reentry channel locations as related to sex-specific risk prediction.

The primary aim of our work was to analyze the influences of both highly detailed anatomical endocardial structures and sex phenotypes on human electrophysiology. To do so, we studied four biventricular, anatomically normal human heart models, reconstructed from high-resolution *ex-vivo* MRI data: one male adult, one male child and two female adults and their smoothed equivalents. The relative influences of sex phenotypes and endocardial sub-structures were studied by applying both phenotypes to all detailed and smoothed geometries and calculating their pseudo-electrocardiogram (pseudo-ECG). In this way we could critically analyze the influences of sex on associated cardiac electrophysiology as well as quantify the errors (root mean square errors) on the pseudo-ECG introduced computationally by neglecting endocardial sub-structures such as trabeculae and false tendons. Further, a full clinical programmed stimulation protocol to assess ventricular tachycardia inducibility was performed on one of these cardiac models after a myocardial infarction was introduced: i.e., into both the detailed and the smoothed geometries. Myocardial infarction information was obtained from a gadolinium-enhanced MRI clinical imaging protocol performed on a different clinical patient.

## Materials and methods

### Human anatomical data

Four biventricular models, two males (one child and one adult) and two females, were segmented from high-resolution MRI data of *ex-vivo* perfusion fixed human hearts. These hearts were obtained from organ donors whose hearts were deemed not viable for transplant thought the local procurement organization, LifeSource. These specimens are part of the Visible Heart^®^ Laboratories human heart library at the University of Minnesota. The uses of these heart specimens for research were given the appropriate written consent, witnessed by LifeSource, from the donor and their families, the University of Minnesota’s Institution Review Board and LifeSource Research Committee. All data has been anonymized from its source. The hearts were cannulated and perfusion fixed under a pressure of approximately 40–50 mmHg with 10% phosphate buffered formalin in order to preserve the hearts in an end-diastolic volume. The images of the fixed hearts were then acquired via a 3T Siemens scanner with 0.44 × 0.44 mm in-plane resolution and slice thickness of 1 to 1.7 mm.

### Anatomical mesh construction

The four hearts utilized for the simulation were selected based on their average mid-cavity LV thickness in order to identify normal, healthy human heart values [10]: 0.63 ± 0.11 cm in male hearts and 0.53 ± 0.09 cm in the female hearts.

**Fig 1:**
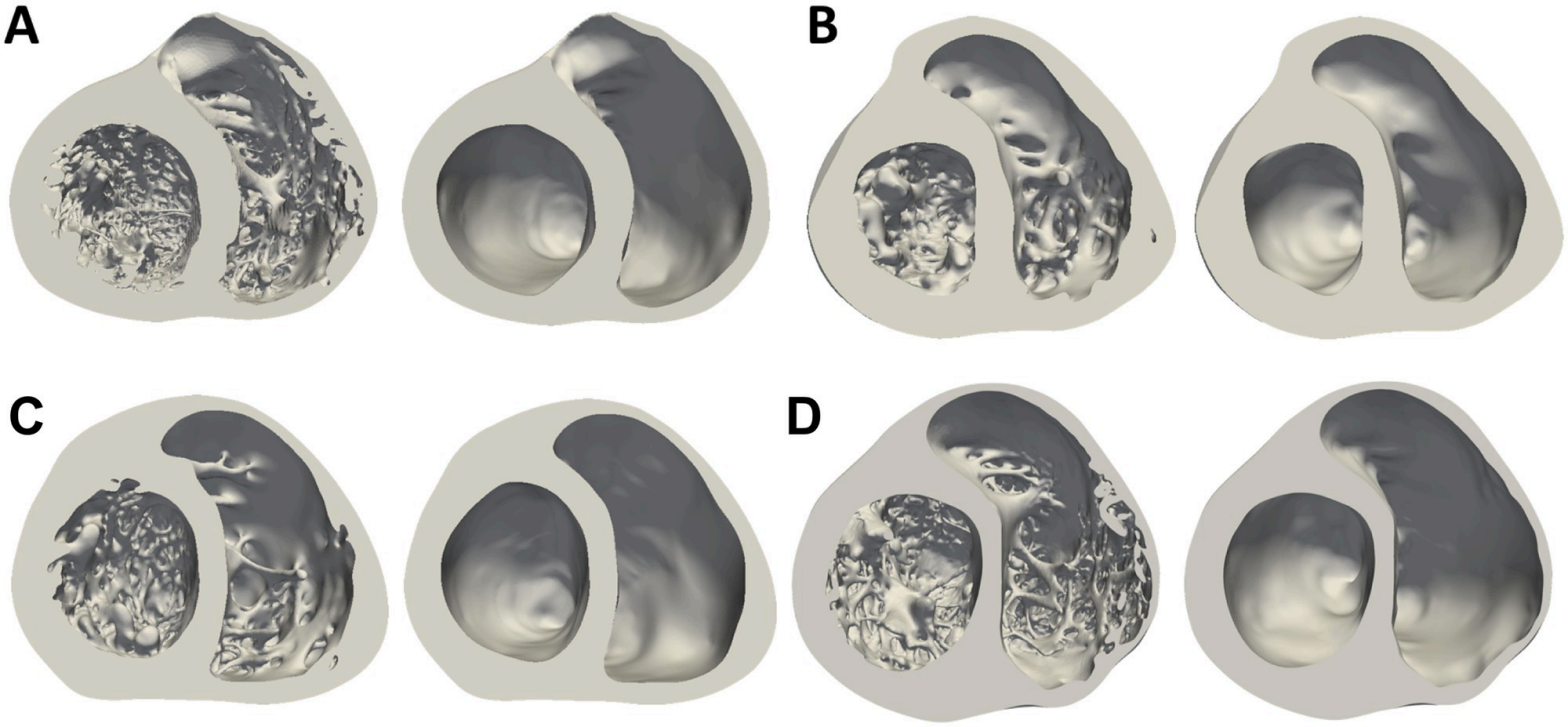
Male (**A-B**) and female (**C-D**) anatomically detailed and corresponding smoothed biventricular geometries. Mid-cavity section.

Corresponding smoothed models were then generated from the detailed ones from the manual delineation of the smoothed endocardial surfaces for each image slice, so to maintain the overall outline of the geometry. The four models and their corresponding smoothed geometries are shown in Fig 1. A detailed description of the methods employed to create the anatomical meshes can be found in the Section S 1.1 in S1 File.

The myocardial volume of each biventricular heart (detailed and smoothed) anatomy is reported in Table 1, along with the volume occupied by the detailed endocardial structures (percentage of trabeculations), the number of long FTs (longer than 1 cm), the average LV thicknesses and tetrahedral-element-mesh information.

**Table 1:** Detailed and smooth biventricular models description.

Heart	A		B		C		D	
Sex	Male		Male		Female		Female	
Geometry	Detailed	Smooth	Detailed	Smooth	Detailed	Smooth	Detailed	Smooth
**Myocardial Volume** [*cm*^3^]	394.2	329.8	199.6	180.5	170.6	155.8	299.2	268
**Trabecular Volume** [%]	16.3		9.6		8.7		10.4	
**N° of FTs ≥ 1cm**	3	//	0	//	4	//	3	//
**Average LV thickness** [*cm*]	0.85		0.83		0.58		0.76	
**Mesh Elements (Million)**	86	72	43	39	37	34	65	58
**Mesh Points (Million)**	14	12	7	6	6	5	11	10

All volumetric meshes had as regular as possible elements with side length between 0.35 mm and 0.4 mm. Wireframe images of the four detailed tetrahedral meshes are shown in the Fig. 1 in S1 File.

### Electrophysiological simulations

The monodomain approximation to electrical propagation was employed as described by Vazquez et al. [11]. Briefly, the reaction-diffusion system to be solved is defined as:

$$\begin{array}{c} \left\{ \begin{array}{c} \frac{d\phi }{dt}+\frac{\partial }{\partial x_{\text{i}}}(D_{\text{ij}}\frac{\partial \phi }{\partial x_{\text{j}}})+\frac{1}{C_{\text{m}}}\left(I_{\text{ion}}\left(\phi ,w,c\right)-I_{\text{stim}}\left(t\right)\right)=0\\ \frac{d(c,w)}{dt}=m_{\text{c},\text{w}}\left(\phi ,w,c\right) \end{array}\right. \end{array}$$ (1)

where *ϕ* is the membrane potential, *C*_*m*_ is the membrane capacitance, *D*_ij_ is the diffusion tensor, I_stim_(t) is the applied current (which initiates activation), I_ion_ is the ionic current term and **w** and **c** are the ionic currents gating variables and the intracellular concentrations, respectively. The O’Hara-Rudy [12] human cardiac ionic model with the modifications suggested in Dutta et al. [13] was employed to compute *I*_*ion*_. The simulation timestep was 10^−2^ms. Before the 3D simulations, the cell model was executed with timestep of 5 × 10^−3^ms until the RMSE between the calcium dynamics of the consecutive beats was lower than 10^−7^mmol, after that the 3D simulations were run for 3 heart beats, when they reached steady state. Each heart beat spent on average of 1.2 h of computational time using 640 cores. Similarly, the programmed stimulus protocol was performed after all models reached steady state during pacing.

**Fig 2:**
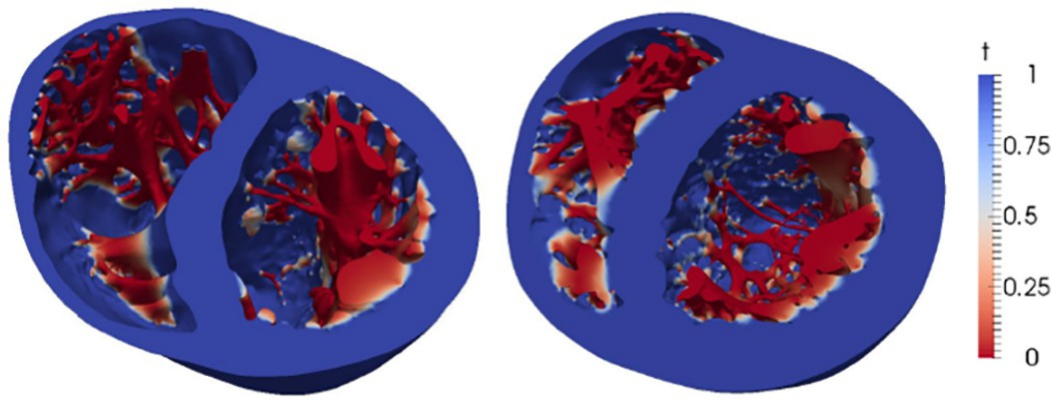
Example of the detection of trabeculae in two different geometries. Function t presents low values in the trabeculae and a smooth transition towards the endocardium.

In this work transmural myocyte heterogeneity was considered by assigning different electrophysiological and cellular properties to the following parts of the cardiac walls: endocardial (inner 30%), mid-myocardial (middle 40%) and epicardial (outer 30%) [12]. The corresponding action potentials are shown in the Fig. 2 in S1 File. Apex-to-base cell heterogeneity was achieved by modifying the conductance of the slow delayed rectifier potassium current *I*_*Ks*_ following a linear gradual decay from apex to base.

Sex phenotypes were incorporated into the O’Hara-Rudy model [12] by modifying all the ion channels sub-unit expression published in [3]. These are reported in the Table 1 in S1 File. Initial conditions for either male or female electrophysiology phenotype were calculated for a cardiac cycle duration of 600 ms.

Finally, a higher diffusion was assigned to a one-element layer on the endocardial surface in order to account for the fast conduction of the Purkinje fibers. The existence of this high conduction layer is important to simulate a physiological conduction velocity in the epicardium and to achieve a normal total activation time. Detailed analyses were carried out with the four models to define physiological diffusion values for both myocardium and endocardium in all anatomies. We hypothesize that there exists approximate common tissue diffusion values across our species. Therefore the sensitivity analysis to obtain these values served the purpose of obtaining an approximate baseline diffusion for normal human electrophysiological function. The responses of the simulations in this study are therefore solely related to the anatomy and the differences in the ion channel kinetics and not related to different tissue properties.

All the electrophysiological simulations were carried out on MareNostrum 4 supercomputer, using the Barcelona Supercomputing Centre (BSC)’s in-house, parallel multi-physics, HPC solver Alya [14–16], with computing time provided by the Red Española de Supercomputacion, RES.

#### Fiber orientation model

The Outflow Tract Rule-Based Model (OT-RBM [17]) was used to assign fibre orientations to all biventricular models. The OT-RBM can deal with complex structures such as trabeculae, papillary muscles, moderator band or false tendons. Myofibers belonging to these structures present a longitudinal orientation along the long axis [18]. The OT-RBM assigns the myofiber orientation to trabeculae by performing two extra steps. First, trabeculae are detected and second, the specific fiber orientation is assigned in these structures. Trabeculae detection is done by calculating the parameter t using the transmural gradient (∇Φ) following the expression in Eq 2.

$$\begin{array}{c} t=\{\begin{array}{cc} \frac{\left\|\nabla \Phi \right\|}{T_{\left\|\nabla \Phi \right\|}}, & if\|\nabla \Phi \|<T_{\left\|\nabla \Phi \right\|}\\ 1, & if\|\nabla \Phi \|\geq T_{\left\|\nabla \Phi \right\|} \end{array} \end{array}$$ (2)

where *T*_∥∇Φ∥_ is a threshold value set as 0.1. An example of the detection of the trabeculae in two different patient geometries can be seen in Fig 2.

**Fig 3:**
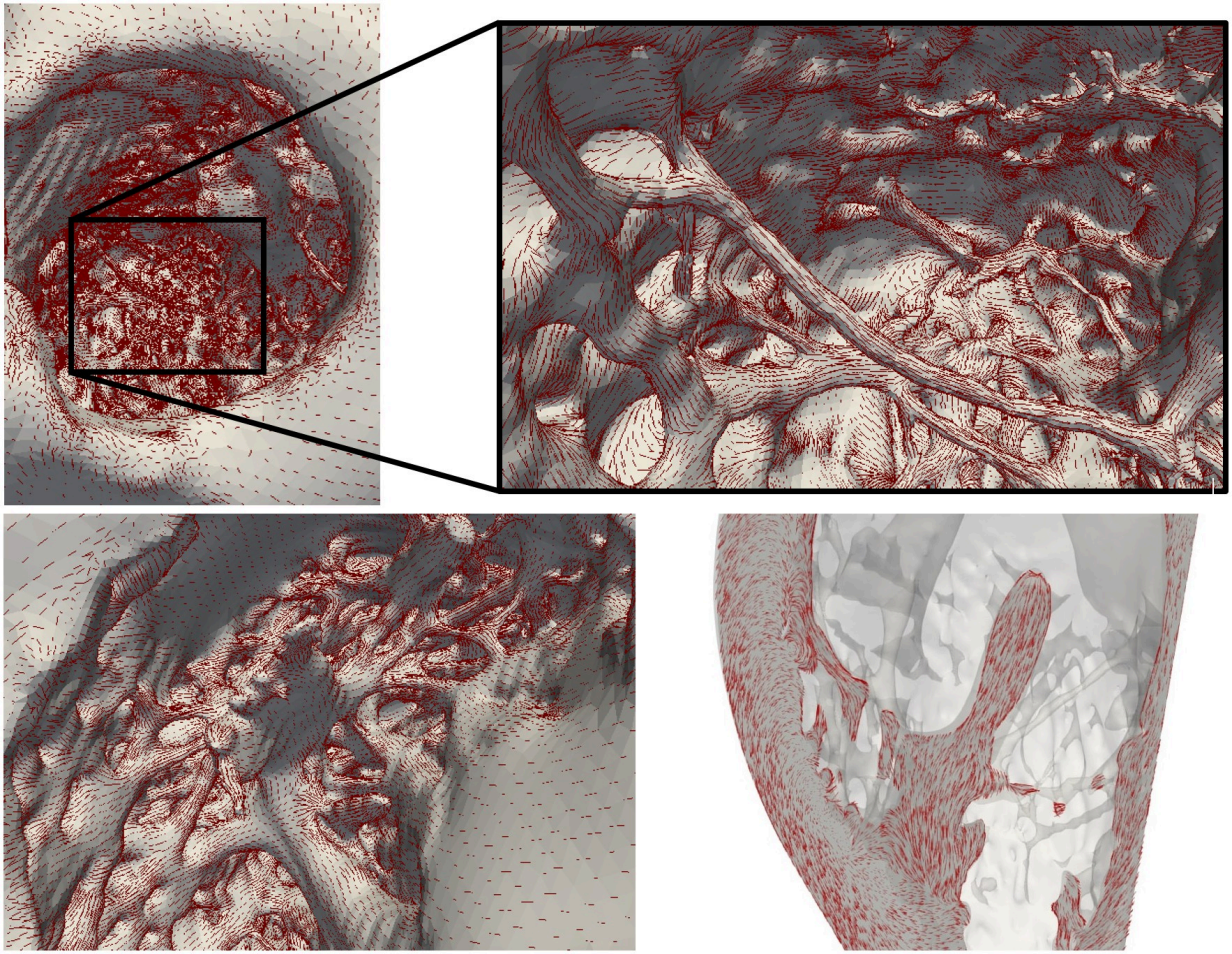
Illustration of the level of detail in the models (geometry A) with fibers, represented by red lines, overlayed. **Top**: Left ventricular endocardium with focus on the longest false tendon. **Bottom**: Right ventricular endocardium. The fiber transition between the trabeculae and papillary muscle is observed in the right panel.

After detecting the trabeculae, fiber orientation is assigned to the trabeculation by means of Eq 3.

$$\begin{array}{cc} \alpha_{trab} & =90\cdot \left(1-t\right)+\alpha_{endo}\cdot t\\ \beta_{trab} & =0 \end{array}$$ (3)

where *α* and *β* are the different angles assigned to the fibers in each mesh node, replicating the angles defined in the original OT-RBM work [17]. The previous expression guarantees a longitudinal direction of the fiber orientation along the trabecula and a smooth transition in the endocardium-trabeculation junction. A close-up of the fibre orientation in both LV and RV of biventricular model **A** is shown in Fig 3. The code to create the fiber orientations resides in a github repository https://github.com/rdoste/OT_RBM.

#### Activation locations

**Fig 4:**
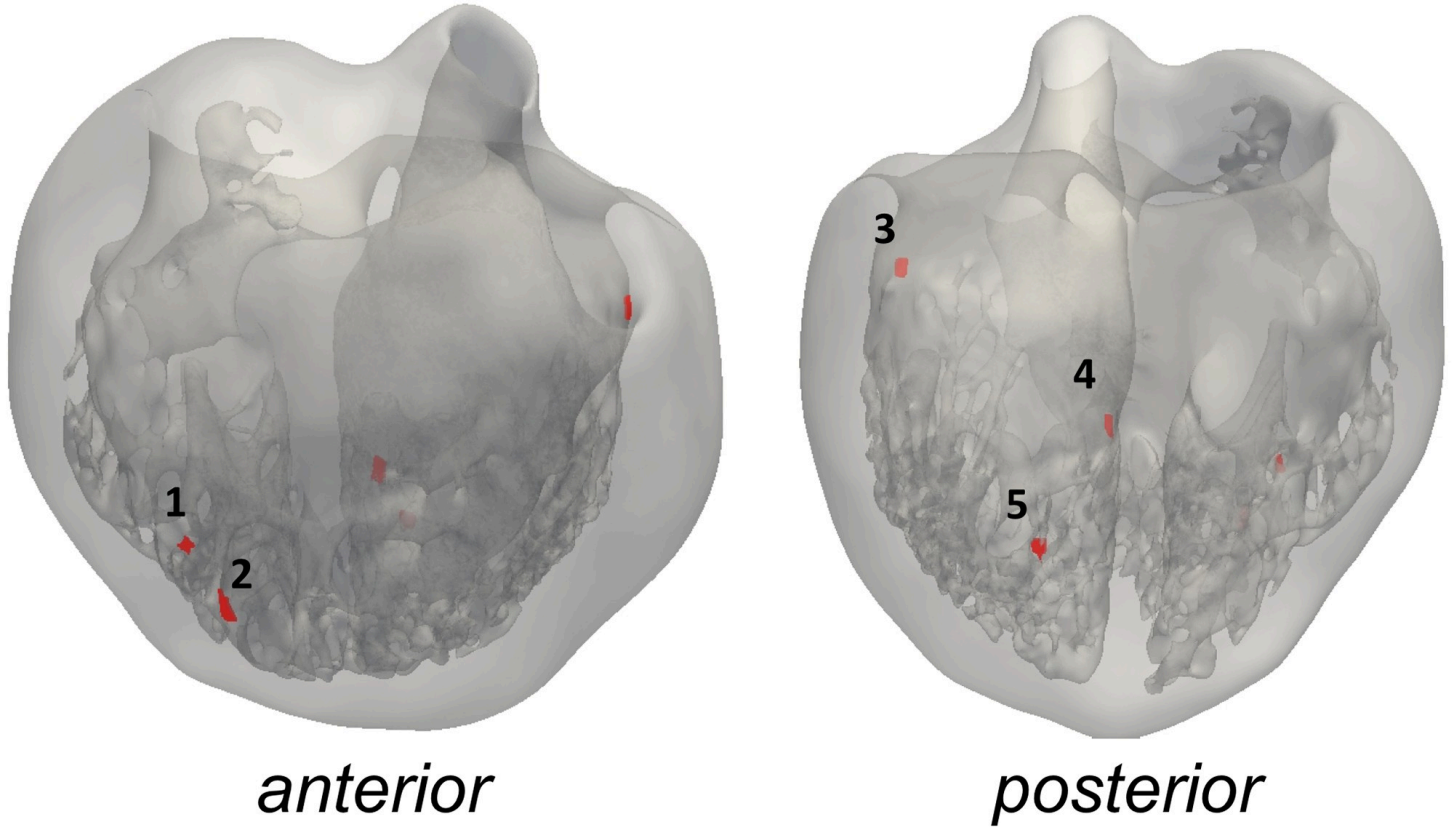
Anterior and posterior view of the geometry C with activation points. Points 1–2 are right ventricular IARs, 3–5—left ventricular IARs).

The location of the activation points within the biventricular cavities was set following the work of Durrer et al. [19]. The initial activation regions (IARs) are displayed in Fig 4 and labeled as follows: **1–2**. two activation areas on the RV wall, near the insertion of the anterior papillary muscle (A-PM) **3**. high anterior para-septal LV area below the mitral valve **4**. central area on the left surface of the septum **5**. posterior LV para-septal area at $\frac{1}{3}$ of the apex-base distance. The same IARs location was used for each detailed/smoothed geometrical couple.

### Parameterization of diffusion

A fast-conducting endocardial surface condition, including both trabecular and FTs network, was used in this work so to simulate the Purkinje fibre network [20, 21]. This fast-conducting endocardial layer is distributed in the lower, apical two thirds of the endocardial cavity, leaving the one third of the basal ring with the diffusion of the bulk myocardial tissue. The same distribution is set on both the detailed and smoothed models. Given that the element sizes of both detailed and smoothed anatomies are the same, the thickness of the layer is preserved and the convergence is ensured. In order to determine the diffusion coefficients to assign to the entire myocardium (*D*_*m*_) and on the endocardial surface (*D*_*e*_), thorough analyses were carried out varying both *D*_*m*_ and *D*_*e*_ in all detailed geometries. Diffusion in both myocardium and endocardium were varied, taking as reference the diffusion value in the work from Bueno-Orovio et al. [22] for the compact myocardium; while for the endocardium we employed a higher diffusion to account for the Purkinje network and obtain physiological values of both total activation times (TATs) and conduction velocity (CVs). The myocardial *D*_*m*_ was assessed by increasing the baseline diffusion value in [22] from 2 times (2*x*) to 7*x*, while the fast conducting layer *D*_*e*_ was assessed in a range of 3*x* to 12*x* of the baseline diffusion until the same diffusions provided physiological TAT and CVs in all anatomies. The selected myocardial and endocardial diffusion values were determined in order to obtain human physiological values of QRS duration and conduction velocities as reported [23, 24] in all geometries (TAT of 100—110 ms for adults and 70—85 ms for the child heart; CV of 41—87 cm/s in adults). This objective was important to discard confounding aspects that could arise due to diffusion coefficient differences between models.

#### Myocardial infarction

A cardiac MRI scan was performed on a patient undergoing electrophysiological studies by our clinical collaborators. Three-dimensional delayed-enhanced acquisitions (1.4 x 1.4 x 1.4 *mm*^3^) were based on a inversion recovery turbo-field echo sequence (IR-TFE). The entire sequence was triggered with a respiratory navigator to compensate for minor volume displacements. Scar segmentation was performed following the methodology of Lopez-Yunta et al. [25]. The scar was registered using ANTS [26] to both the smooth and detailed geometries of one case, heart *D*, as shown in Fig 12, preserving the transmurality of the scar on the new anatomy as much as possible. Details can be found in the supplement. The border zone, or heterogeneous scar region, was parameterized following the experimental measurements of [27], with an isotropic diffusion equivalent to the transverse diffusion employed for the normal myocardium. The dense scar was modelled as an inactive (non-excitable) material with an extremely small isotropic diffusion equivalent to 10% of the normal extracellular transverse diffusion used in [28], in the fiber direction, equivalent to a conductivity of 0.024*mS*/*mm* and 90% lower in the transverse direction.

#### Tachycardia inducibility protocol

The clinical protocol for risk stratification of Mark Josephson [29] was employed to assess the ventricular tachycardia risk on the smooth and detailed anatomy with male and female action potential phenotypes. The selected S1 basic cycle length for the programmed stimulation protocol was set to 400 ms. Three stimuli locations were employed: apex of left ventricle (LV Apex), right ventricle (RV Apex) apex, and right ventricular outflow tract (RVOT). Once a sustained tachycardia was obtained, the data was analysed and the subsequent stimulation location was run.

### Data analysis

To assess the influence of anatomy and sex phenotype on electrophysiology simulations, all heart anatomies were simulated with both male and female phenotypes in order to dissect these sex-specific differences from the anatomical variations. A two-tailed unequal variance t-test (Welch t-test) was used to compare groups of measurements and the null hypothesis was rejected at 0.05.

The pseudo-ECG, was calculated by locating each biventricular model within a torso and recording the cardiac electrical activity at three field coordinates (or “electrodes”) positioned at the approximate location of the right arm (RA), left arm (LA) and left leg (LL). The pseudo-ECG was calculated as an integral over the spatial gradient of the transmembrane voltage within the cardiac tissue. The pseudo-ECG is calculated without considering the tissue conductivity within the torso between the heart and the leads [30, 31]. A detailed explanation on the calculation of the pseudo-ECG can be found in the Section S 1.4 in S1 File.

The following quantities of interest were analysed:

***Trabecular volume*** was calculated as the percentage change between the volume of the detailed (*V*_*d*_) and the one of the smoothed (*V*_*s*_) biventricular geometries: 100 ⋅ (*V*_*d*_ − *V*_*s*_)/*V*_*d*_.

***Total activation time*** (*TAT*) was calculated as the time at which the biventricular geometries were fully depolarized at V ≥ 0 mV. Additionally, we quantified the time required to reach the 10% (*TAT*10) and 90% (*TAT*90) of the activation of the entire tissue volume. *TAT*_*Gdiff*_ was calculated as the difference between the *TAT* of the detailed minus the smoothed geometries of the same sex. Similarly *TAT*_*Gdiff*10_ and *TAT*_*Gdiff*90_ are calculated as the difference between *TAT*10 and *TAT*90 respectively of the detailed minus the smooth anatomies of the same sex.

***Electrical dyssynchrony index*** (*EDI*) was calculated as the standard deviation of the local activation time of the complete geometry. *EDI*_*Gdiff*_ was calculated as the difference of *EDI* in the detailed minus the smoothed anatomy for the same sex.

***Apparent conduction velocity*** (*CV*) was calculated as the mode of the conduction velocity distribution in sinus rhythm on the epicardial surface of the geometries. *CV*_*Gdiff*_ was calculated as the difference of *CV* in the detailed minus the smoothed anatomy for the same sex. It highlights the differences in the epicardial apparent conduction velocity observed due to the existence of alternate propagation pathways and gender phenotype differences.

***QRS complex duration*** (*QRS*), of the pseudo-ECG, was calculated as the time between the onset of the complex (equal to the start of the stimuli), to the the last inflection point of the QRS complex, where the voltage of the three leads (LI, LII and LIII) cross the baseline value.

***QT interval duration*** (*QT*) of the pseudo-ECG, was calculated as the time between the onset of the complex, at the beginning of the stimulation time, to the the last inflection point of the T wave, where the voltage of the three leads (LI, LII and LIII) reach the baseline.

***QT interval duration difference*** (*QT*_*diff*_) was calculated as the difference between the QT interval duration between the female (*QT*_*f*_) and male (*QT*_*m*_) phenotype on the same geometry: *QT*_*diff*_ = *QT*_*f*_−*QT*_*m*_. *QT*_*Gdiff*_ was calculated as the difference between the QT interval duration between the detailed and the smoothed geometries.

***QRS duration difference*** (*QRS*_*diff*_) was calculated as the difference between the QRS duration between the female (*QRS*_*f*_) and male (*QRS*_*m*_) phenotype on the same geometry: *QRS*_*diff*_ = *QRS*_*f*_ − *QRS*_*m*_. *QRS*_*Gdiff*_ was calculated as the difference of the QRS duration between the detailed and smoothed geometries.

***Pseudo-ECG root-mean-squared difference (RMSD)***was calculated between the pseudo-ECG of the smoothed and detailed geometries and for both male and female phenotypes.

The activation pattern of the detailed models was qualitatively analyzed and compared to the smoothed cases.

***Sustained Ventricular Tachycardia*** was characterised by three or more reentrant full heart activations during the S1-S2-S3-S4 pacing protocol. Any other arrhythmic events are defined as reentries that do not sustain for more than 2 beats.

## Results

### Diffusion analyses

After conducting a thorough study on all four biventricular geometries, a single set of diffusion coefficients for the fast-conducting endocardium and myocardial tissue were identified for all hearts. The objective was to find an approximate normal value of diffusion that can reproduce normal human electrophysiological behaviour in various detailed anatomies (TAT of 100—110 ms for adults and 70—85 ms for the child heart and CV of 41—87 cm/s in adults). The diffusion values obtained for the myocardium were 5.8 ⋅ 10^−3^*cm*^2^/*ms* in the fiber direction and 1.9 ⋅ 10^−3^*cm*^2^/*ms* in the transverse and normal directions. For the purkinje layer, the diffusions obtained were 1.7 ⋅ 10^−2^*cm*^2^/*ms* in the fiber direction and 5.8 ⋅ 10^−3^*cm*^2^/*ms* in the transverse and normal directions. The membrane capacitance and surface to volume ratio correspond to the ones reported for the O’Hara-Rudy [12] cardiomyocyte model.

### Electrophysiology simulations

**Fig 5:**
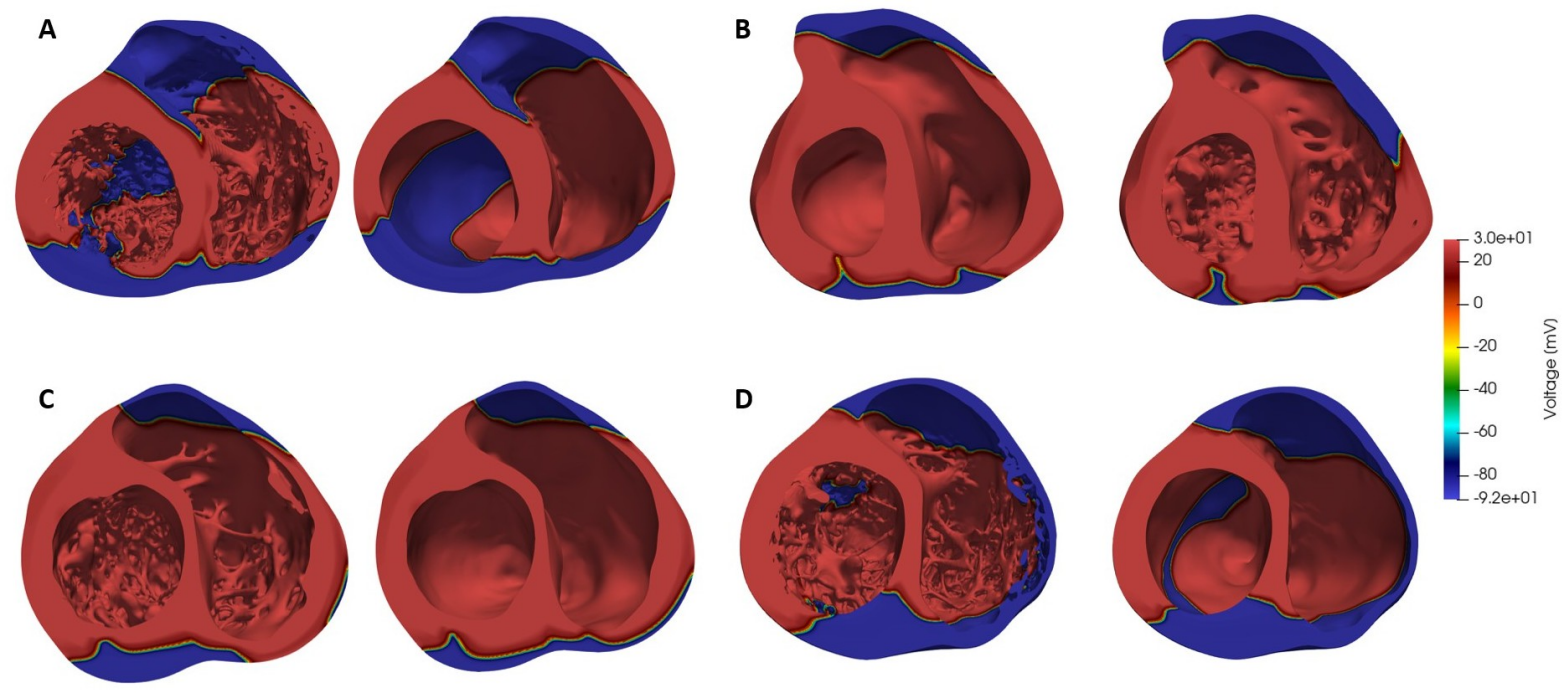
Depolarization wave distribution at 60 ms, male phenotype.

**Fig 6:**
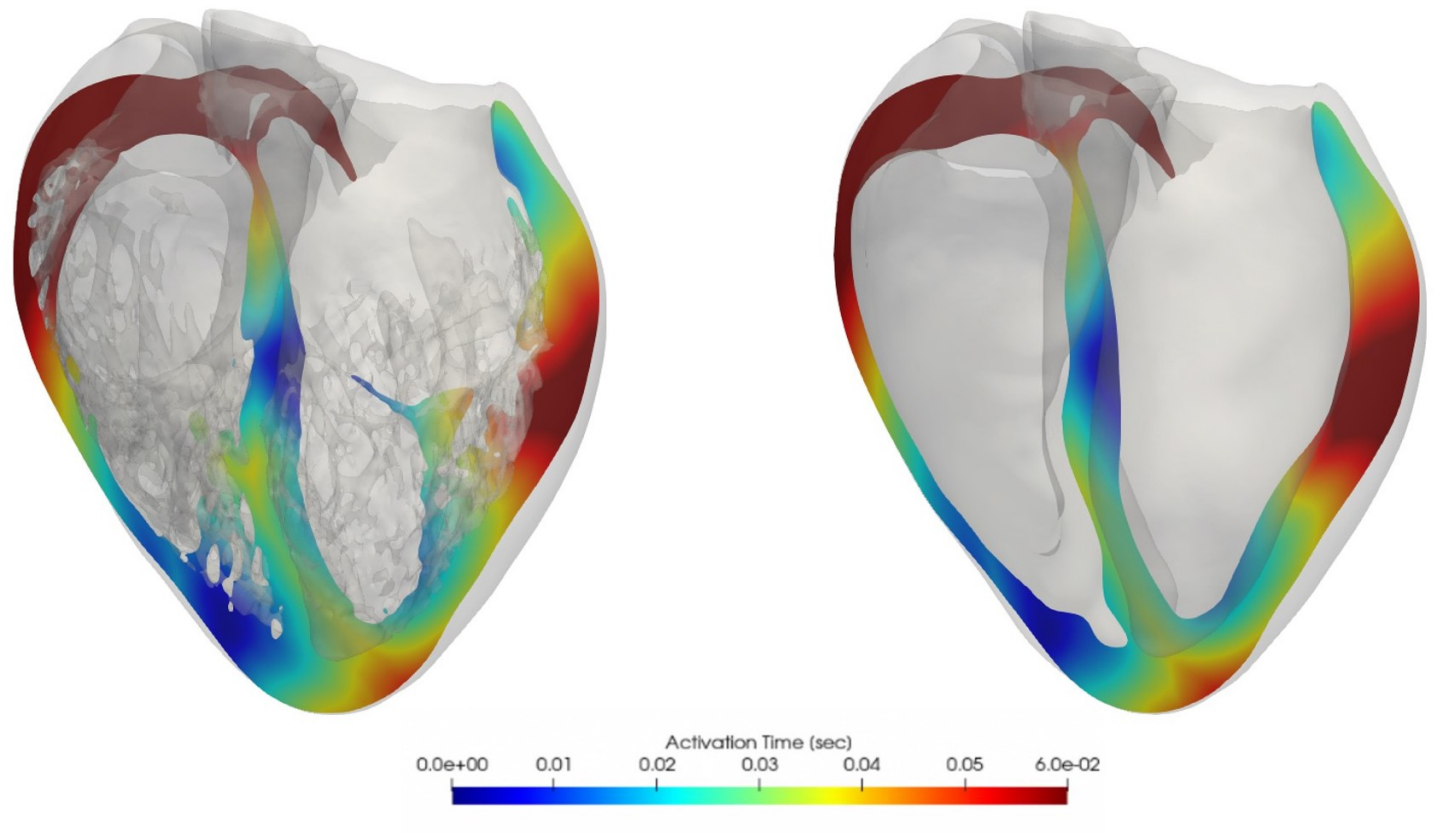
Cross-sectional slices of ventricular isochrone activation maps, smoothed (left) and detailed (right) heart **D** comparison, male phenotype.

**Fig 7:**
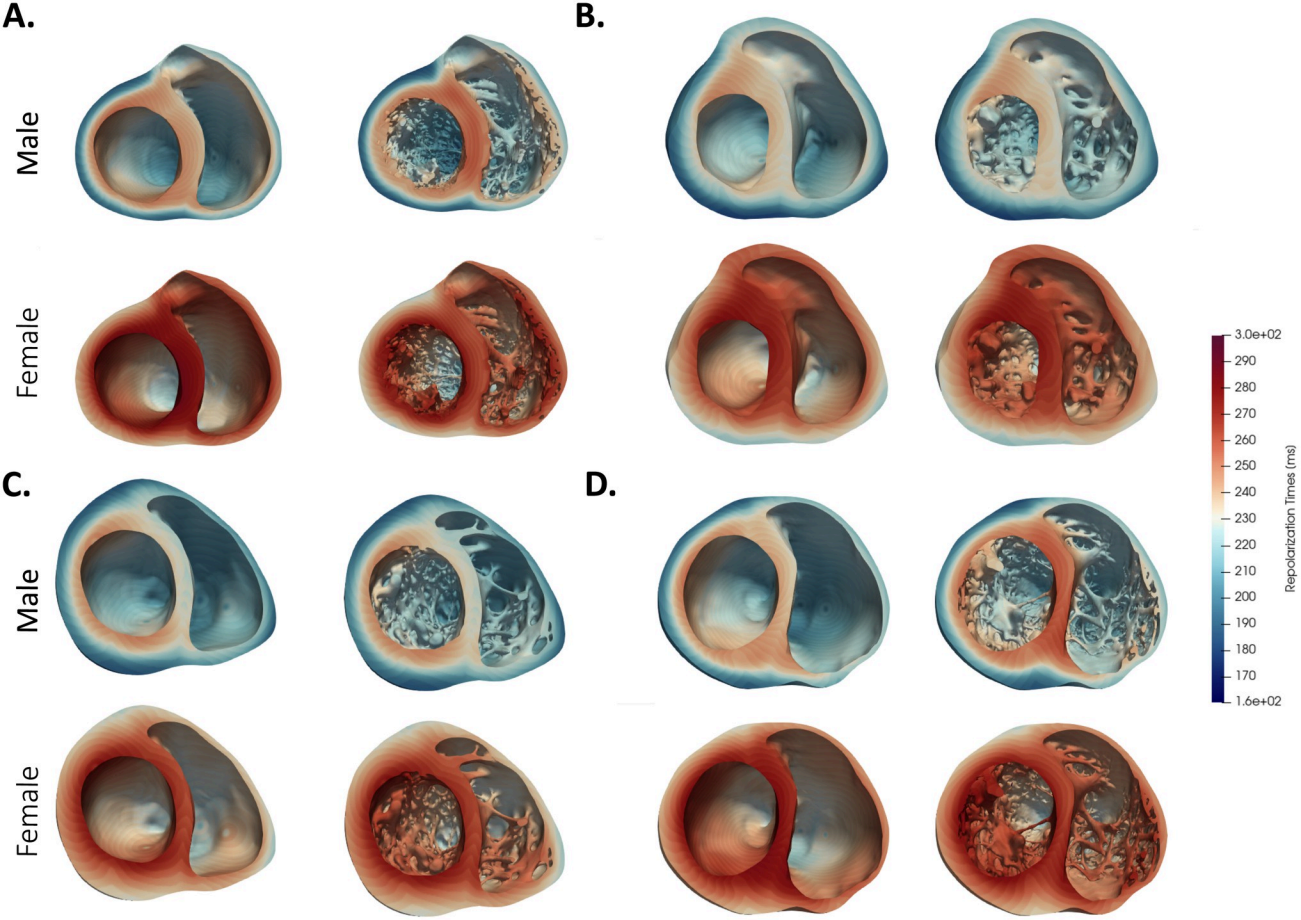
Repolarization map of each heart (**A-D**) for both sexes and details levels. Repolarization time is defined as the time of 90% repolarization of the AP.

Results from electrophysiological simulations of the four detailed and smoothed biventricular models with both male and female phenotype are listed in Table 2. As can be seen, female phenotype prolonged the QT interval in all cases. Noticeable local differences on the activation pattern between the detailed and smoothed geometries can be observed in Fig 5. Local distribution of the depolarization wave is shown at 60 ms for both detailed and smoothed cases with male phenotype. Isochrone maps of activation of every model can be observed in the S1 File. False tendons provide a fast activation bridge that change the activation pattern in the detailed geometries as observed in Fig 6. Lastly, on Fig 7 the local repolarization map for each heart, geometry and sex are shown. Both, sex and anatomical complexity induces appreciable differences in the repolarization times both in length and dsitribution.

**Table 2:** Electrophysiological simulation results.

Heart	Geometry	Sex	QRS [ms]	QRS_*diff*_ [ms]	CV [cm/s]	QT [ms]	QT_*diff*_ [ms]	TAT [ms]	TAT_10_ [ms]	TAT_90_ [ms]	EDI [ms]
A	Detailed	M	97	-1	90	349	42	104.3	12.3	66.8	20.1
		F	96		90	391		104.3	12.3	66.8	20.1
	Smooth	M	99	2	80	339	28	99.8	13.8	71.3	21.3
		F	101		80	367		100.0	13.8	70.8	21.3
B	Detailed	M	95	0	81	339	37	96.6	9.3	64.8	20.2
		F	95		86	376		96.5	9.3	64.8	20.2
	Smooth	M	83	1	88	335	63	84.4	10.8	59.8	18.1
		F	84		86	367		84.4	10.8	59.8	18.1
C	Detailed	M	90	3	83	337	27	92.5	7.3	58.3	18.8
		F	93		84	364		92.6	7.3	58.3	18.8
	Smooth	M	93	1	85	329	30	94.4	9.8	60.3	19
		F	94		86	359		94.5	9.8	60.3	19
D	Detailed	M	104	4	85	357	34	119.2	11.3	67.8	21.3
		F	108		83	391		119.3	11.3	67.8	21.4
	Smooth	M	99	5	86	359	40	120.6	11.8	71.3	22.6
		F	104		86	399		120.5	11.8	71.8	22.6

### Pseudo-ECGs

**Fig 8:**
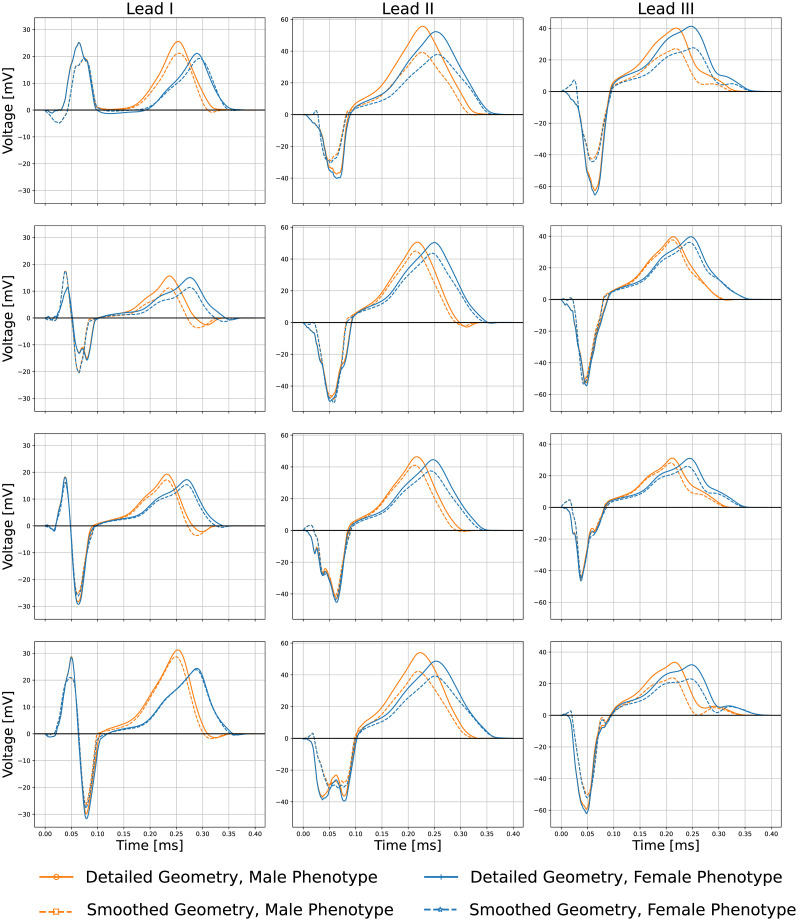
Lead I, II and III pseudo-ECG results for smoothed/detailed male (**A-B**) and female (**C-D**) geometries with both male/female phenotypes.

Fig 8 shows the obtained pseudo-ECG for Lead I, II and III, for smoothed and detailed male (**A-B**) and female (**C-D**) geometries respectively, with both phenotypes. QT prolongation is observed in all female phenotypes cases, as expected, however an increase on the action potential duration did not linearly translate into the same increase of QT-interval durations. Table 3 shows the RMSD between the pseudo-ECG of the smoothed and detailed geometries for both male and female phenotypes. It is noticeable that the differences between the smoothed and detailed geometries can affect both QRS segment morphology and T wave magnitude. The T wave is the most different feature in the pseudo-ECGs between sexes. Interestingly, heart C, which has the smallest trabecular volume, also has the smallest difference between the detailed and smoothed pseudo-ECG RMSD.

**Table 3:** Root mean square difference between the pseudo-ECG waveform of the detailed- and smoothed-geometry simulations on both female and male sex phenotypes.

Heart	Phenotype	Lead I (mV)	Lead II (mV)	Lead III (mV)
**A**	Female	2.3	6.6	7.2
**B**	Female	2.6	4.0	2.5
**C**	Female	1.1	3.3	2.7
**D**	Female	1.5	5.2	4.9
**A**	Male	2.6	7.1	6.9
**B**	Male	2.7	4.0	2.3
**C**	Male	1.2	3.1	2.4
**D**	Male	1.9	5.9	5.2

### Ventricular tachycardia inducibility

A total of 126 simulations were done to reproduce the programmed stimulation protocol commonly performed in the clinical setting (S1-S2-S3-S4) to stratify the risk of postinfaction cardiac arrest. The arrhythmic events throughout the inducibility study are detailed in Table 4. The results show a high number of arrhythmic events (48%) in the male phenotype, detailed heart, while the smooth geometry of the same sex phenotype shows a 14% of events. For the female phenotypes of the detailed and smoothed geometries only a 6% and 1% of arrhythmic events, respectively, occurred throughout the entire protocol. The female phenotype simulations elicited a sustained ventricular tachycardia (VT) with an RV apex stimulus on both, the detailed and smooth geometries. Ventricular tachycardia occurred only with an LV apex stimulus in the case of the detailed, male geometry while sustained ventricular tachycardia occurred on both RV Apex and RVOT stimulus locations in the case of the smooth male geometry.

**Table 4:** Arrhythmic events including reentries and sustained VTs. The number of events (E) are shown along with the total number of simulations (S) for each programmed stimulus protocol at each location.

Stimulus Location	RV Apex	RVOT	LV Apex	Total
**Events/Simulations**	E/S	E/S	E/S	E/S
**Detailed Female**	2/13	0/12	0/8	2/33
**Smooth Female**	1/11	2/10	0/8	3/29
**Detailed Male**	4/10	7/15	6/10	17/35
**Smooth Male**	2/10	1/9	1/10	4/29

**Fig 9:**
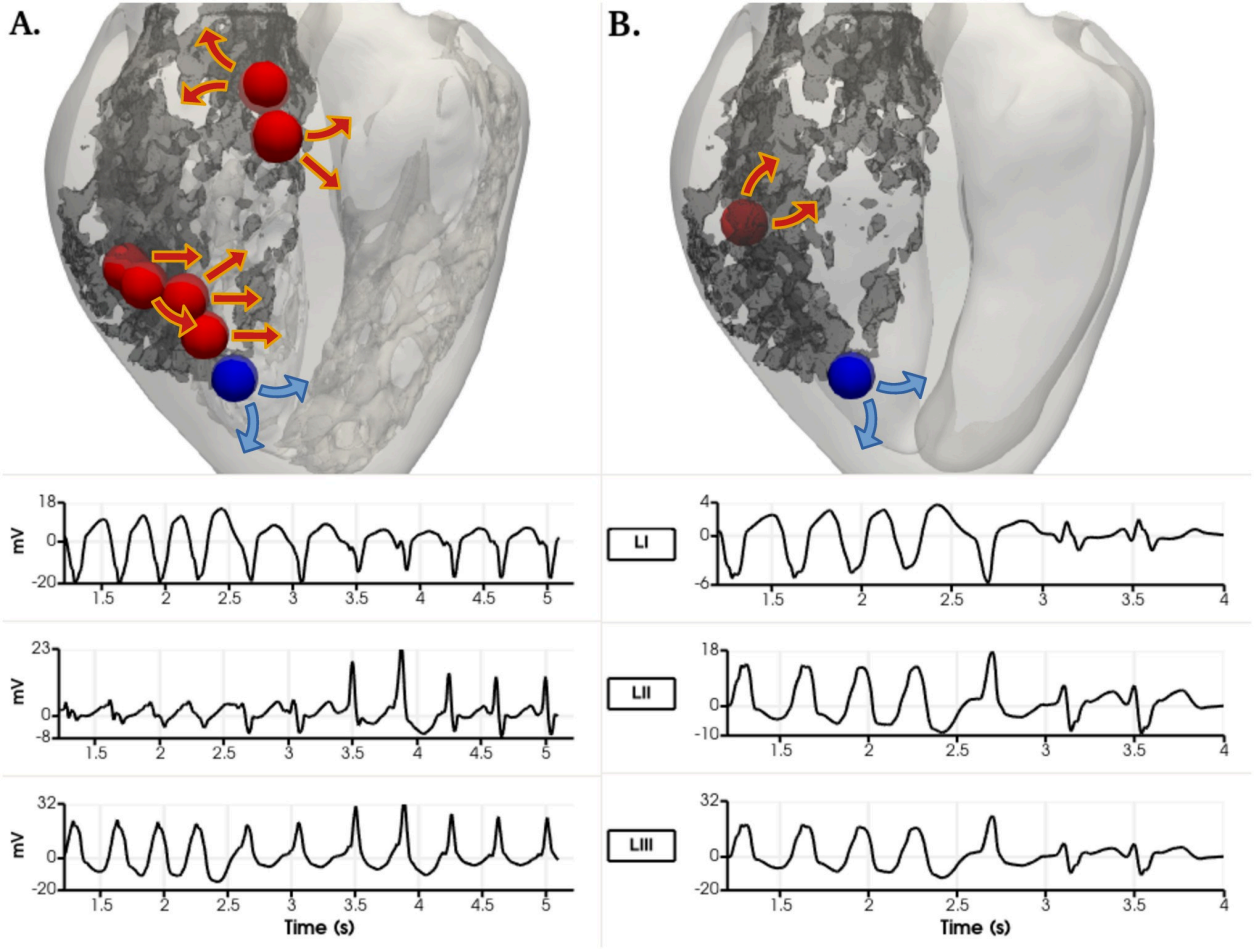
Ventricular tachycardia reentry channels in the female phenotype simulations with right ventricular apex stimulus location at S1 = 400, S2 = 300, S3 = 300, S4 = 300 ms. View from the epicardial posterior side of the biventricular geometry. Dense scar marked in dark grey. Arrows show the propagation wavefront of each reentry. A. Detailed geometry. B. Smoothed geometry. Their corresponding pseudo-ECG is shown below for the LI, LII and LIII leads. The sphere in blue shows the exact same reentry channel on detailed and smoothed simulations.

**Fig 10:**
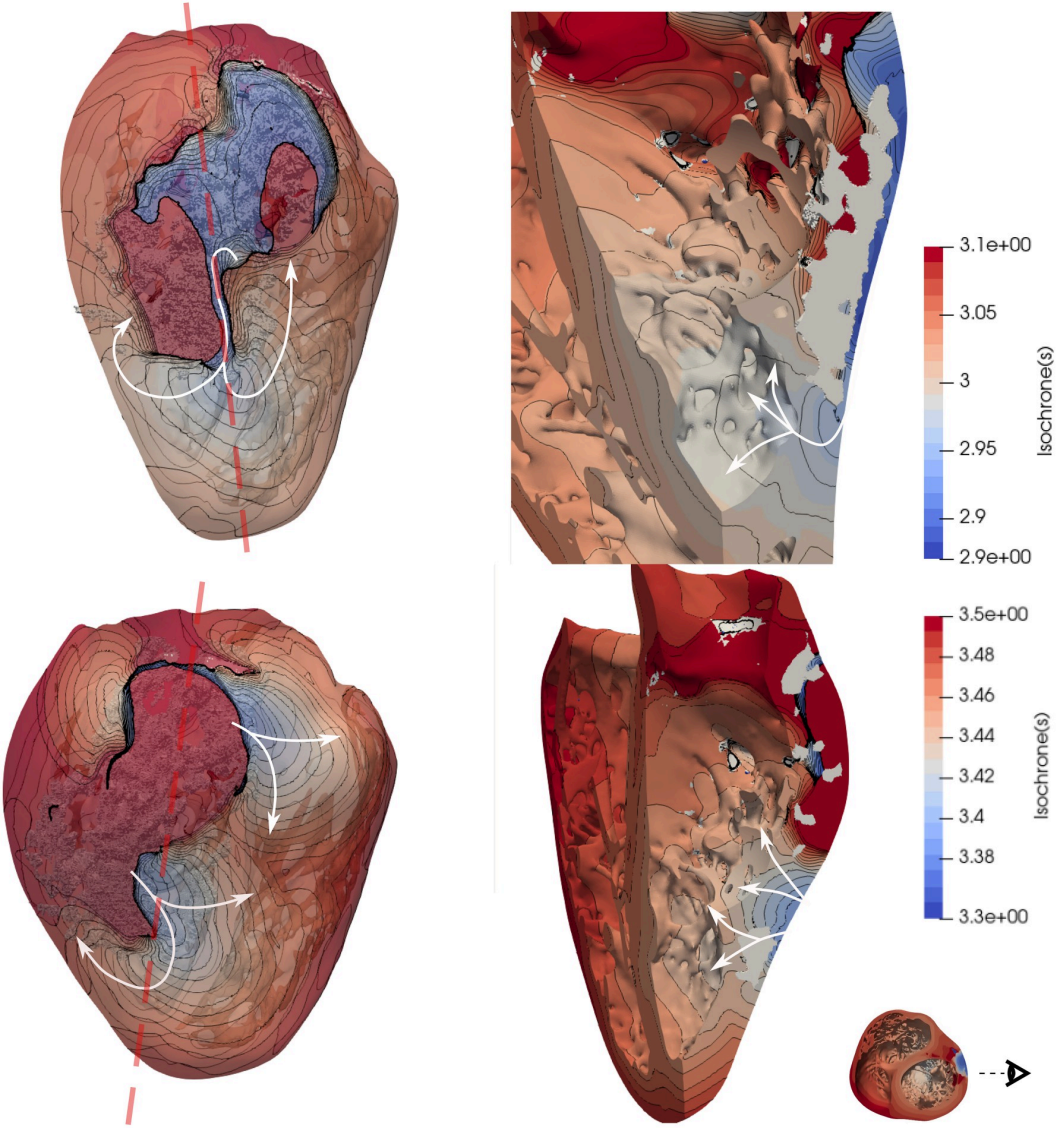
Isochrone map of the ventricular tachycardia obtained from the female detailed simulations with RVApex stimulus location (same as in Fig 9A) at two different time intervals, as shown in the color bars. Dense scar is observed in white color. The papillary muscle was removed for ease of visualization on the right panels. S1 Video in S1 File shows this VT.

**Fig 11:**
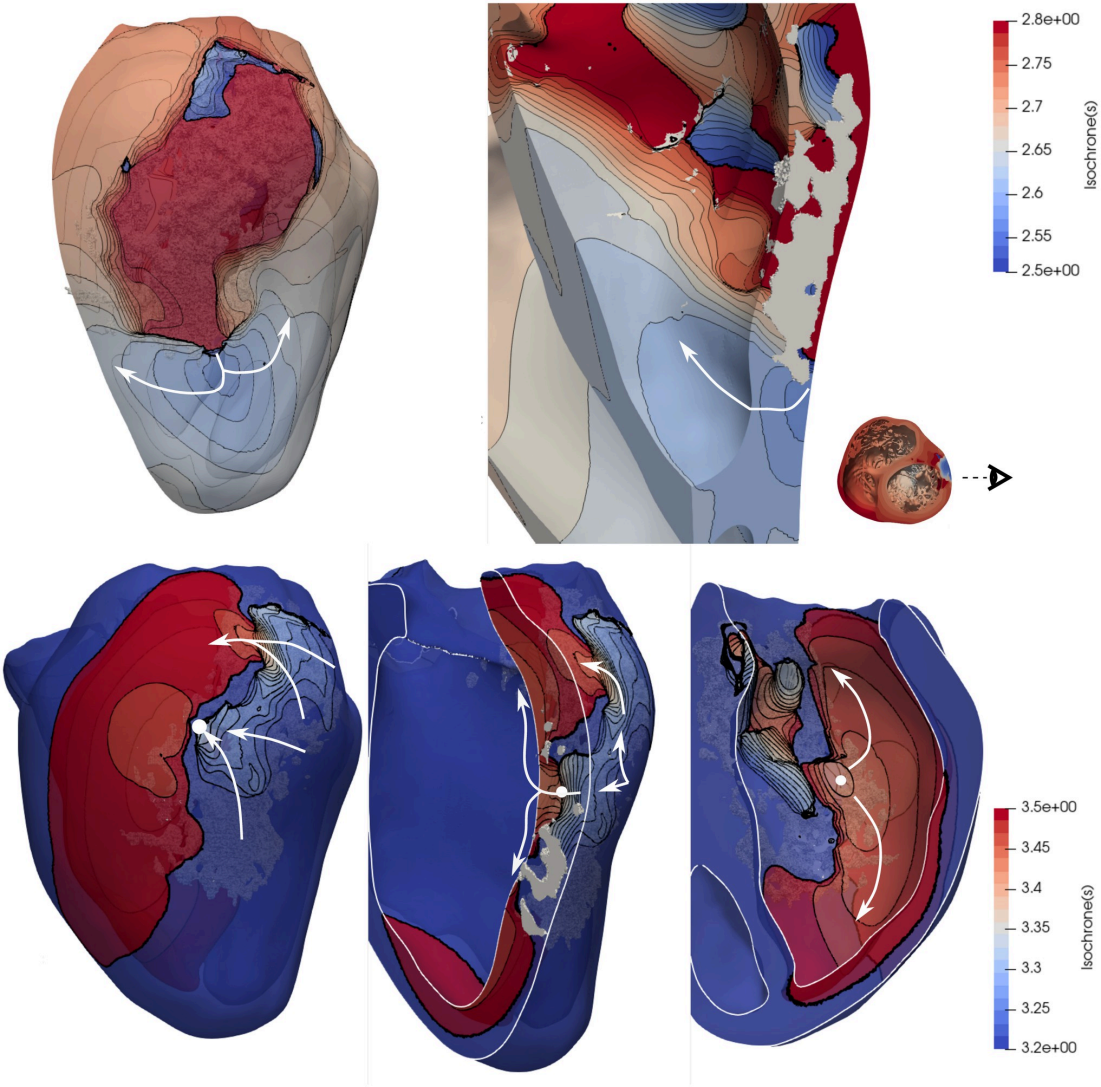
Isochrone map of the ventricular tachycardia obtained from the female smooth simulations with RVApex stimulus location (same as in Fig 9B) at two different time intervals shown in the color bars. Dense scar is observed in white color. A white circle shows a transmural channel, which provides a different reentry pathway to the shown in Fig 10. S2 Video in S1 File shows this VT.

**Fig 12:**
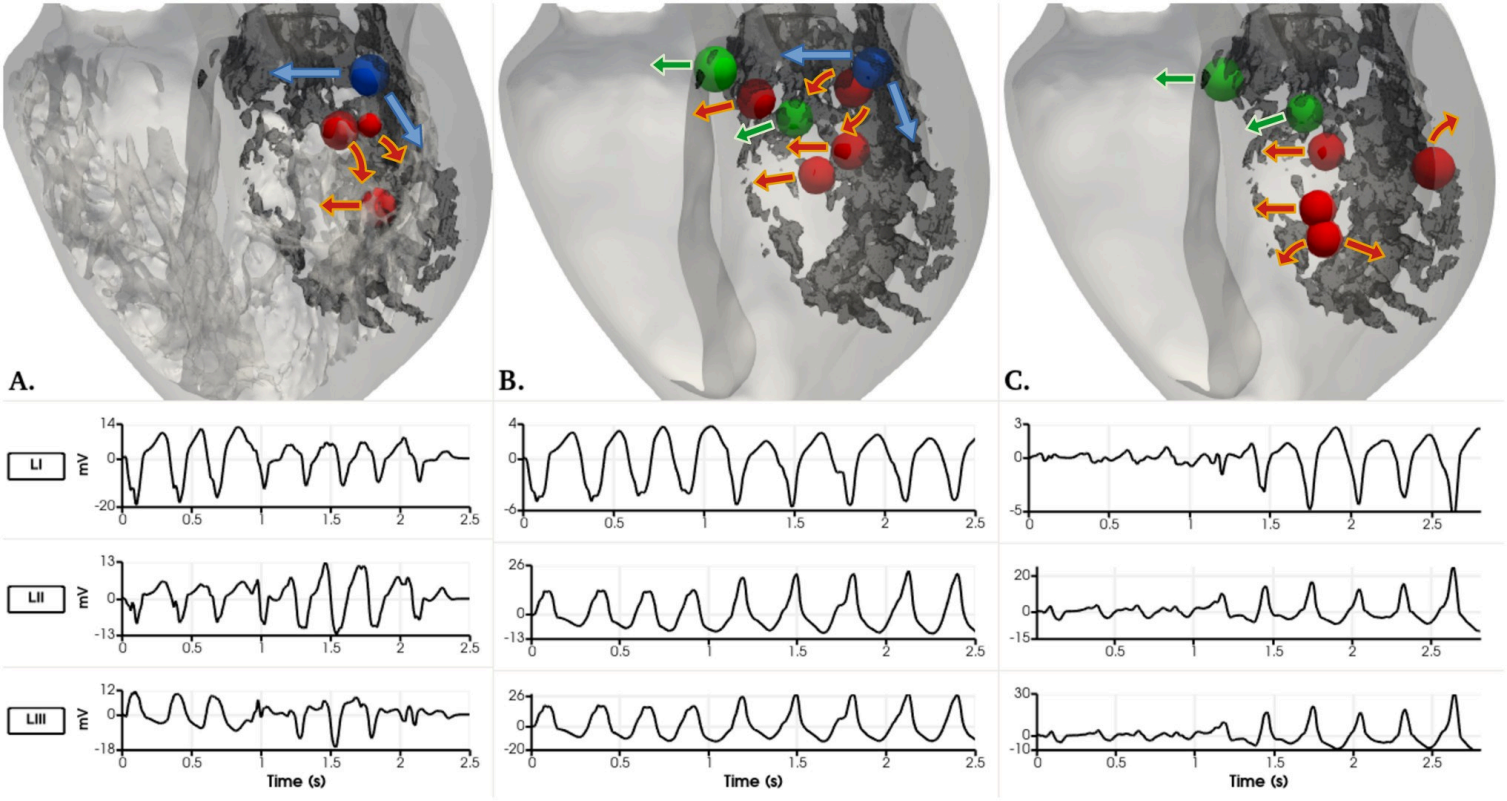
Detailed and smoothed male VT simulations. The dense scar is seen in dark grey colour on all three geometries for reference. View from the mid longitudinal plane towards the posterior side of the heart. Arrows show the propagation wavefront of each reentry. A) Four reentry channel locations on the detailed male geometry with an LV apex stimulation shown as spheres. B) Seven reentry channel locations on the smoothed male geometry with an RV apex stimulation shown as spheres. The blue sphere on A and B corresponds to the same location. C) Six reentry channel locations on the smoothed male geometry with a RVOT stimulus shown as spheres. The green spheres on B and C correspond to the same channel location. Underneath each geometry, the three pseudo-ECG leads recorded for each case.

Each VT was analyzed in order to locate the channels that enabled the tachycardia to sustain. Figs 9 and 12 show the comparisons between geometries and phenotypes for each simulation with a sustained VT. The VT on both female phenotype geometries occurred at an S1 = 400, S2 = 300, S3 = 300 and S4 = 300. Interestingly, on both geometries the initial reentry location is exactly the same, and it is marked as a blue sphere in Fig 9. The channel locations for the subsequent reentries enabling the VT are completely different, and marked with red spheres. The two female VT simulations provide different reentry cycle lengths. The detailed geometry presents reentries of variable timings: 363, 420, 410, 420, 380, 350 and 370 ms and all are located at the posterior epicardial wall. An isochrone map of four reentry locations can also be observed in Fig 10, were the reentry occurs transmurally and through the epicardial tissue. The epicardial channel locations vary at each wave reentry. The smoothed geometry reentrant cycle lengths were 300, 700 and 400 ms and only one of the reentry channels was located at the epicardium. Similarly, an isochrone map of different reentry locations can also be observed in Fig 11, were the reentries occur through the epicardial, endocardial and transmural tissue.

Sustained VT was obtained on the detailed, male phenotype simulations at S1 = 400, S2 = 270, S3 = 260, S4 = 240 ms using the LV apex stimulus location. The tachycardia is of a more regular nature with cycle lengths of 270, 270 and 290 ms as observed in Fig 12. The reentry channel for the initial three re-entrant beats is located at the LV posterior wall, approximately in the center of the scar. Interestingly, a trabeculae (small, red sphere in Fig 12A.) created a pathway to create a reentry channel, which created a fast pathway of re-activation towards the apical septum region through the endocardial structures. The last reentry channel is located towards the base of the posterior LV wall, shown in blue.

The sustained tachycardia obtained in the smoothed, male geometry occurred during RV apex stimulation at S1 = 400, S2 = 260, S3 = 250, S4 = 240 ms. Seven reentry channels were identified during a 2.8 second simulation. Two are located at the septum, two more channels on the posterior LV wall. The VT ultimately sustains through a channel at the basal location shown in blue in Fig 12B. Interestingly the two reentry channel basal locations shown in blue on the detailed (Fig 12A.) and smooth (Fig 12B.) anatomies are the only channels that correspond between the two simulations. In the case of the smoothed, male geometry VT generated using the RVOT stimulation location at S1 = 400, S2 = 260, S3 = 230, S4 = 240 ms, six channels are located to create the reentrant waves: on the basal section of the septum, the basal section of the LV free wall, and various reentry beats arising at the posterior LV wall, approximately below the papillary muscle. Interestingly, only two channels, after an RV apex and RVOT stimuli on the smooth male geometries shown in green in the Fig 12B and 12C. coincide. All pseudo-ECG recordings are provided for detailed analysis. All of the male phenotype simulations elicited reentries at the mid-myocardial or endocardial transmural regions. A video of each VT simulation can be found in the S1 File.

## Discussion

The cardiac ventricular endocardium of humans are highly trabeculated and complex, such anatomical characteristics are often ignored in ventricular electrophysiology studies. This is the first time, to the best of our knowledge, that all small endocardial sub-structures with cross-sectional area ≥ 1 mm^2^, were utilized in human cardiac electrophysiology simulations; here we modeled four detailed, biventricular, anatomically normal human hearts. Previous studies have develop detailed hearts of one rabbit as in [5] or a human one like in the work of Connolly et al [8], but none of them provides a complete human heart representation with this level of detail, with 4 different geometries and analysing the sex influence on the VT risk. Sex phenotype has been another characteristic often neglected, which was also considered in our study. It is important to note that all bio-markers were analyzed using statistical tools even though the sample number is small (four to eight samples). Obtaining high resolution ex-vivo imaging data of anatomically normal human hearts is extremely difficult, and therefore only four anatomies were employed. Most normal donor human hearts are preferably and understandably employed primarily for transplantation, hence their little availability for research. Yet, the authors also understand there are limitations to the statistical power of these results, but the analyses of an even larger data set is an active part of our ongoing work.

### Diffusion parameterization

The objectives for performing thorough diffusion coefficient analyses were to obtain the approximate values that would produce physiological values of TATs and CVs on all the detailed geometries. The selected diffusion coefficients were employed to establish the baseline condition for all our human heart simulations. This was specifically done to eliminate any confounder results due to different diffusion coefficients among hearts. It is however, important to note that the optimal diffusion coefficient estimated corresponded to our assumption of a fast-endocardial diffusion: i.e., that was needed to compensate for the fact that we did not include Purkinje networks. When no fast-endocardial condition was included, it was impossible to obtain normal TATs and CVs (within measured ranges) in all of our human heart simulations.

### Sex differences in detailed and smoothed geometries

The two-sample Welch t-test provided significant differences on the QTs relative to sex; i.e., between both male and female detailed and smoothed geometries (p = 0.003 and p = 0.013, respectively). It was expected that the female phenotypes would present longer QT intervals [3]: the average QTs prolongation was 35.00 ms on the detailed geometries and 40.25 ms on the smoothed ones. Moreover, as it is observed, in Fig 7 the female phenotype produces longer repolarization times as expected. These differences are generated by the reduced expression of repolarization key ion channels [3] (i.e, potassium ion channels) mostly, in endocardial and epicardial female cells, having as a final outcome this prolonged repolarization times. However, it is clear that the approximate APD lengthening due to sex phenotype is not the same for all anatomies, as observed in the *QT*_*diff*_ marker in Table 2. Tissue interactions within the heart anatomies produce an anatomy- and sex- dependent QT-lengthening.

Sex-specific phenotypes may play a role for the simulation of cardiac arrhythmias, where QT-interval values influence the triggering and/or sustaining of arrhythmias; i.e., particularly for reproducing the cycle length of a patient-specific arrhythmia.

#### Differences in pseudo-ECG

The pseudo-ECG calculations cannot be directly compared to clinically measured ECG data because of the limitations of our methodology (the lack of the solution of the propagation through the torso). Furthermore, small registration errors (rotation or translation) can impact the lead waveforms. In addition, we had no functional data for these subjects and the activation sequences employed were generalization from the data measured by [19]. Our main uses of the pseudo-ECGs were to quantify the variations between the anatomies and phenotypes. Results show that QRS complexes followed almost the same profiles for both male and female phenotypes, but were different when compared between smoothed and detailed ventricular geometries. The predicted differences of the QRS complexes between detailed and smoothed geometries correspond to the different regional activation patterns observed in the absence of endocardial structures. Furthermore, it is also noticeable that on some leads, the QRS wave appears fractionated in the detailed anatomies, feature which disappears in the smoothed anatomies. It is clear that QRS fractionation is produced by the shortcut pathways created by false tendons. The T waves showed differences, as can be observed from Fig 8; particularly a decrease on the peak magnitude in the smoothed anatomies, and the QT interval prolongation in the female sex simulations. The reduction of the T-wave magnitude within the smoothed geometries in comparison to the detailed anatomies was particularly observed in hearts **A** and **D**; interestingly, these are the hearts with a larger ventricular volume. The smoothed geometries introduced an average RMS error on each pseudo-ECG lead of approximately 2.0, 4.9 and 4.3 mV for both male and female phenotypes (see Table 3). The measured errors between the smoothed and detailed anatomies seem to be directly correlated to the trabecular volume in each heart.

Hence we can conclude that the absence of endocardial structures modifies the QRS complex (eliminates fractionation) and decreases the T-waves magnitude; which are directly related to the subject-specific trabecular volume and structures and the activation and repolarization sequences on any computed lead.

### Differences between detailed and smoothed geometries

Anatomical differences produced QRS variations that do not correlate to the trabecular volume of each case as quantified in Table 5. The largest QRS differences occurred on heart **B**, where the smooth geometry decreased the QRS duration in both male and female phenotypes. Instead, the QRS duration in heart **A** was longer in the smoothed geometry for both male and female phenotypes. Heart anatomies **C** and **D** had sex-dependent QRS change variations between detailed and smooth geometries.

**Table 5:** Electrophysiological simulation results. Quantification of differences between detailed and smoothed geometries. Markers are shown as the difference between the detailed minus the smoothed geometries.

Heart	Sex	QRS_*Gdiff*_ [ms]	CV_*Gdiff*_ [cm/s]	QT_*Gdiff*_ [ms]	TAT_*Gdiff*_ [ms]	TAT_10*Gdiff*_ [ms]	TAT_90*Gdiff*_ [ms]	EDI_*Gdiff*_ [ms]
A	M	-2	10	10	4.5	-1.5	-4.5	-1.2
	F	-5	10	24	4.3	-1.5	-4	-1.2
B	M	12	-7	4	12.2	-1.5	5	2.1
	F	11	0	9	12.1	-1.5	5	2.1
C	M	-3	-2	8	-1.9	-2.5	-2	-0.2
	F	-1	-2	5	-1.9	-2.5	-2	-0.2
D	M	5	-1	-2	-1.4	0.5	-3.5	-1.3
	F	4	-3	-8	-1.2	0.5	-4	-1.2

The QRS-interval estimation has a high clinical relevance, and its measurement can be easily affect by the clinical expertise. The TAT marker is, however, easily measured within our simulations. This marker is therefore useful for detailed analysis of the models. As reported in Table 5, there is no clear correlation of TAT_*Gdiff*_ (average 3.35 ms, p-value = 0.3) between detailed and smooth geometries; on anatomies *A* and *B* the TAT_*Gdiff*_ is longer, opposite to anatomies *C* and *D*. Nonetheless, there seems to be a shorter TAT_*Gdiff*10_ (average -1.5ms, p-value = 0.06) and TAT_*Gdiff*90_ (i.e, -1.12 ms, p-value = 0.32). Trabecular tissue helps to reach and activate faster and in a more synchronous way the ventricular tissue, supported by the reduction of TAT_10_, TAT_90_ and EDI. Moreover, larger regional differences in activation times, caused by overall different propagation paths (lack of shortcuts) of the depolarization waves, were found in the smoothed cases, although there were no consistent effects on global TATs. As one can observe in Fig 5, indeed, smoothed geometries were characterized by different propagation paths of the depolarization waves as compared to the detailed anatomic cases mostly due to FTs and trabeculae. Similar findings were found on the simulations done by *Bishop et al*. [5] and the ones from *Connolly et al*. [8], in which both highlighted the potential shortcuts and pathways that these ventricular substructures might induce on the depolarization and repolarization waves. This is clearly visible in Fig 6, where cross-sectional slices representing the activation times at the level of the main FT are shown. FTs provide a clear propagation shortcut for the depolarization and for the repolarization (See Fig 7D). CV_*Gdiff*_ was only slightly faster in the detailed geometries, but heart *A* was the exception.

Lastly, QT-interval values were generally longer in detailed anatomies, however this effect did not occur in all hearts. Interestingly, the QT_*Gdiff*_ in the detailed anatomies did not correlate to the myocardial nor the trabecular volume of each heart. Furthermore, the QT-interval differences between anatomies were also sex-dependent.

It is clear that ventricular substructures aid in the activation propagation by inducing a more synchronous and faster propagation, activating a bigger volume of tissue in shorter times. The inclusion of these cardiac details in cardiac electrophysiological simulations might have a large impact in scenarios of electrical disturbance with ventricular assynchronous activation.

### Impact of sex and geometry on tachycardia inducibility

The results provided evidence that there is an impact of both endocardial structures and sex phenotypes on the prediction of reentry channels that create and sustain ventricular tachycardia. The reentry pathways identified were often different, with only one reentry location being the same between geometries for both male and female phenotypes. It was highly interesting that the induced VTs occurred at different stimuli locations between the smooth and detailed geometries. The refractory periods were also highly important, they differ according to the employed geometry and/or locations of the stimuli. The results regarding the inducibility of tachycardia may provide physiologically relevant information regarding the risk of females to VT at different hormonal stages during their life time. It is highly interesting that while the male phenotype produced a much higher percentage of arrhythmic events throughout the programmed stimulation protocol than the female phenotype, the VT induced in the female detailed anatomy sustained for a longer period of time. This work provided evidence of the importance of the personalization of the approximate repolarization time on patient-specific VT simulations. Furthermore, it provides evidence of the limitations regarding the prediction of reentry channels using anatomical models with smooth endocardium. A reliable methodology for the prediction of reentry channels should be thoroughly investigated for patient-specific applications.

## Limitations

One of the primary limitations of the present study was the somewhat small number of human hearts analyzed in this study. The study focuses on anatomically normal human hearts, which are generally and preferably, transplanted; therefore the access to high-resolution, anatomically normal human heart images are rare. It is therefore a fact that using a larger population, our findings regarding both the diffusion coefficient analyses and sex/anatomical differences will provide higher statistical power.

A second limitation that should be noted was the lack of a 1D Purkinje network coupled to the 3D myocardium. We used instead a fast-endocardial condition and we provided sensitivity analyses to estimate the optimal myocardial and endocardial diffusion coefficients. Although it is possible to extract and represent computationally the free-running Purkinje system in our geometries, this becomes more tricky to create on top of the complex endocardial structures. Building an artificial purkinje network on top of these anatomies would also represent an approximation far from histologically measured Purkinje networks running within human ventricles and would provide a higher variability of the inputs of the models. The optimal way to validate the simulations in terms of the activation systems would be to image the Purkinje system from high-resolution contrast enhanced micro-CT of human hearts [32]. Work is ongoing to do so, but this may take years to accomplish. Furthermore, the VT inducibility would also be impacted by the presence of a 1D Purkinje network. The current simulation assumes that there is retroactive activation through the fast-activating layer after reentry, which might not be fully representative of a physiological scenario. The consistency of this limitation within the simulations allows for their comparison, but makes difficult the comparison to clinical data. Therefore it is an important limitation that we did not optimize the models to reproduce a standard clinical electrocardiogram. All four anatomies were spatially located within a generic single torso geometry using the exact same methodology regardless of how each signal correlated to clinical electrocardiographic recordings. The same applied to the activation locations. The pseudo-ECG recordings presented cannot be clinically validated. A methodology that could allow a more precise mapping of activation locations between anatomies could have been implemented, as has been done previously to create an independent frame of reference between anatomies [33]. Therefore, further optimization to reproduce clinically relevant ECG is ongoing work, in line with published work [34]. Finally, only approximate cardiac electrophysiologic quantities of interest were analyzed in this current study; i.e., there was no consideration relative to the effects of electro-mechanical coupling. In other words, the lack of mechano-electric feedback in our described simulation, may provide different results versus fully coupled electro-mechanical simulations. Nonetheless the results presented in this paper provide novel insights relative to the effects of sex phenotypes as well as and the roles of trabeculae and FTs on the overall cardiac electrophysiologic behavior.

## Conclusion

Electrophysiological simulations of four human biventricular heart biventricular models were presented with high geometrical details, which to the best of our knowledge, has not been reported yet in the electrophysiological simulation literature. This study uniquely represents some of the first insights in human cardiac electrophysiological simulations relative to assessing the roles of both endocardial substructures and sex phenotypes. This work provides strong evidence that neglecting either endocardial anatomical details or cell-level sex characteristics for cardiac electrophysiology simulations may lead to erroneous results. Our work indentified a significant QT-interval increase in the detailed female human heart phenotypes; i.e., shown also by the pseudo-ECGs results, coinciding with the reported QT prolongations in phenotypically female hearts (independently of the geometry). QRS durations were also significantly longer in the female phenotypes. Therefore the pseudo-ECG patterns are subject to variations due to both anatomy and sex. Additionally, our simulations suggest that the absence of trabeculations introduces regional differences in the propagation pathways relative to the depolarization wavefront. The presence of FTs were considered to shortcut the signal propagation leading to differences in local activation patterns. Finally, it was shown that the levels of endocardial details and differences in the sex phenotypes also will likely affect the arrhythmic risks and conduction channel locations: i.e., during the in-silico, clinical assessments of tachycardia inducibilities.

Our future work includes the analyses of larger datasets, in order to verify statistical significances, in addition to including the effects of the mechano-electric feedback on clinically relevant pathological scenarios, thus both aiming to increase the accuracy of cardiac computational model predictions for their clinical translation.

## Supporting information

S1 File Supporting methodology. (PDF)
