# Supplementary material for: Ventricular anatomical complexity and sex differences impact predictions from electrophysiological computational models
Source: PLoS One. 2023 Feb 13;18(2):e0263639. doi: 10.1371/journal.pone.0263639 (PMC9925004; doi:10.1371/journal.pone.0263639)
Supplement: S1 File — (PDF) [file pone.0263639.s001.pdf]

Ventricular anatomical complexity and sex differences impact predictions from electrophysiological computational models

Pablo Gonzalez-Martin[2], Federica Sacco[1,3], Constantine Butakoff[2], Ruben Doste[4], Carlos Bederian[5], Lilian K Gutierrez Espinosa de los Monteros[6], Guillaume Houzeaux[1], Paul A. Iaizzo[7], Tinen L. Iles[8], Mariano Vazquez[1,2], Jazmin Aguado-Sierra[1*]

1 Barcelona Supercomputing Center, Barcelona, Spain
2 ELEM Biotech S.L., Barcelona, Spain
3 Physense, Department of Information and Communication Technologies, Universitat Pompeu Fabra, Barcelona, Spain
4 Department of Computer Science, University of Oxford, Oxford, UK
5 Instituto de Física Enrique Gaviola - CONICET, Córdoba, Argentina
6 Centro Nacional de Investigaciones Cardiovasculares Carlos III, Madrid, Spain
7 Visible Heart® Laboratories, Department of Surgery and the Institute for Engineering in Medicine, University of Minnesota, Minneapolis, USA
8 University of Minnesota Medical School, Minneapolis, USA

* jazmin.aguado@bsc.es

S1 Supporting methodology.

S1.1 Anatomical mesh construction.

The software Fiji[1] [1] was used to carry out high resolution MRI segmentation, with the maximum entropy-based thresholding algorithm [2]. The images were characterized by excellent contrasting gray scales, which permitted thresholding to achieve biventricular heart models with highly detailed endocardiums (endocardial structures included were ≥ 1 mm^2 in cross-section). The reconstruction of four biventricular meshes including detailed endocardial structures was accomplished utilizing Seg3D [3] marching cubes algorithm. The obtained surface meshes were uniformly remeshed with the platform Remesh [4] and volumetric tetrahedral meshes were generated using ANSYS ICEM CFD (ANSYS® Academic Research Mechanical, USA). Wireframe images showing the element distribution within each heart anatomy are shown in Fig. 1.

S1.2 Electrophysiology solver.

Finite Element Method (FEM) and Finite Difference Method (FDM) were used for the space and time discretisation of the electrical activation potential propagation equation:

$$\left(\frac{M}{\Delta t} + \theta G\right)\Delta\phi + M^{\mathrm{d}} I_{\mathrm{ion}} = -G\phi^{\mathrm{n}} \tag{1}$$

where $\Delta\phi = \phi^{\mathrm{n}+1} - \phi^{\mathrm{n}}$ is the unknown difference between two time steps, M is the mass matrix, G is the electrophysiological stiffness matrix, and θ determines whether the time integration scheme is first order explicit, Forward Euler ($\theta = 0$), first order implicit, Backward Euler ($\theta = 1$) or second order implicit, Crank-Nicholson ($\theta = 0.5$).

The discrete equation is then solved using a first order Yanenko operator splitting as follows:

[1] https://imagej.net/Fiji

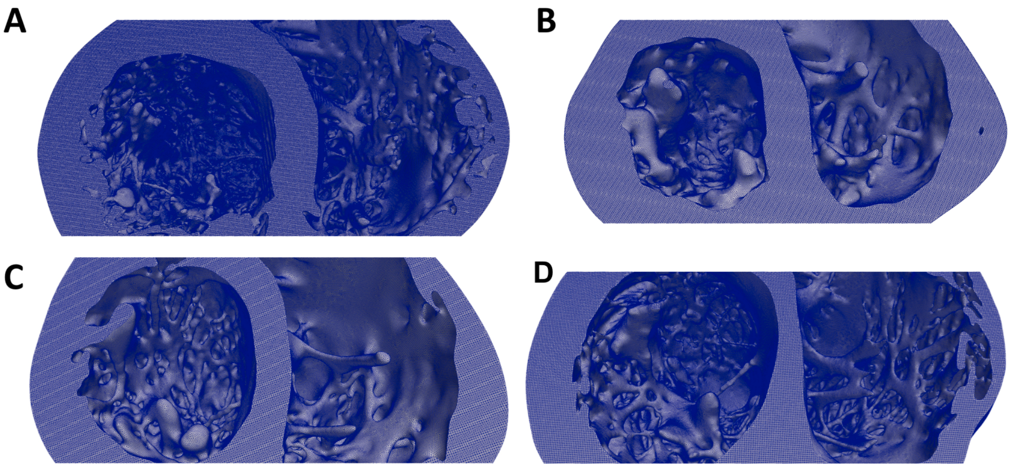

Fig 1. Male (**A-B**) and female (**C-D**) anatomically detailed biventricular wire-frame meshes, mid-cavity sections. Elements are tetrahedral and regularly sized throughout the whole anatomy. Element size is the same in all four anatomies.

$$\text{Cell model}(\Delta\phi^*) : \frac{\Delta\phi^*}{\Delta t} + I_{ion}(\phi) = 0 \quad (2)$$

$$\text{Tissue model}(\Delta\widetilde{\phi}) : \left(\frac{M}{\Delta t} + G\right)\Delta\widetilde{\phi} = -G\phi^* \quad (3)$$

$$\text{Update}(\phi^{n+1}) : \phi^{n+1} = \phi^n + \Delta\phi^* + \Delta\widetilde{\phi} \quad (4)$$

where the *Cell model* is solved explicitly using a Forward Euler scheme and the *Tissue model* is solved implicitly with either a Backward Euler or a Crank-Nicholson scheme s described by [5].

S1.3 Sex phenotypes.

Table 1. Ion channel conductances ratios relative to male endocardial cell. No change on mid-myocardial cells was applied.

Ion channel	Endocardium		Epicardium	
	Male	Female	Male	Female
I_{Ks}	1	0.83	1.04	0.87
I_{Kr}	1	0.79	1.09	0.875
I_{K1}	1	0.86	0.98	0.74
$I_{to,s}$	1	0.64	0.6	0.26
I_{NaK}	1	0.79	0.94	0.7
I_{pCa}	1	1.6	0.88	1.6
I_{up}	1	1.15	1.42	1.97
$Calm$	1	1.21	1.01	1.41

S1.4 Pseudo-ECG calculation.

The calculation of the pseudo-ECGs derived from the calculation of the unipolar potentials (ϕ_e) as follows:

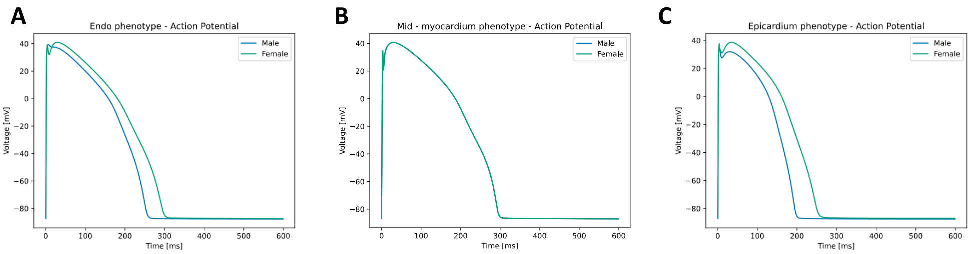

Fig 2. Effect of sex-specific ion channel kinetics on the action potential celltypes employed to define transmural heterogeneity in this study. From left to right: endocardial, mid-myocardial M and epicardial celltypes coloured by sex (green: female; blue: male). Note that the M-cell was not modified with sex-specific ion channel kinetics given that no experimental information regarding sex differences exist.

$$\phi_\mathrm{e}(x',y',z') = \int D[-\nabla V_\mathrm{m} \cdot (\nabla \frac{1}{r})]dx + \int D[-\nabla V_\mathrm{m} \cdot (\nabla \frac{1}{r})]dy \quad (5)$$
$$+ \int D[-\nabla V_\mathrm{m} \cdot (\nabla \frac{1}{r})]dz$$
$$r = [(x-x')^2 + (y-y')^2 + (z-z')^2]^{\frac{1}{2}} \quad (6)$$

where D is the diffusion tensor at every Gauss point, ∇V_m is the spatial gradient of the transmembrane potential and r is the distance from a source point (x,y,z), which represents a point on the heart geometry, and a field point (x',y',z'), which represents the position of one of the electrodes used to calculate the pseudo-ECG (LA, RA, LL). Electrical potential difference was defined as the difference in electric potential between two electrodes; these potential differences are represented as "leads". There is always one exploring (positive) and one recording (negative) electrode. In this way, a propagation wave going towards the exploring electrode produces a positive wave and vice versa. The three leads were defined as:

$$Lead_I = \phi_{LA} - \phi_{RA}, \quad (7)$$
$$Lead_{II} = \phi_{LL} - \phi_{RA}, \quad (8)$$
$$Lead_{III} = \phi_{LL} - \phi_{LA} \quad (9)$$

S1.5 Numerical verification.

Numerical verification is essential, and it was performed following an electrophysiology N-version verification benchmark [6]. To that end, a very similar methodology was implemented with the O'Hara-Rudy cell model. Briefly, a slab was employed, where elements were subdivided to analyse spatial and temporal convergence of conduction velocity [7]. The problem was setup as follows. The slab length of the initial mesh was divided in three sections, where the three different celltypes were defined (endo, M and epi), the first layer of elements on the endocardial edge of the slab were assigned higher diffusion coefficients to replicate the fast activation of the conduction system employed in this study. The diffusion coefficients employed were 5.8 cm^2/s in the longitudinal and 1.9 cm^2/s in the transverse direction. Results show that the convergence of the element size 0.348 varies only by 3% from an element side length of 0.0175 cm, as observed in Table 2. Data is plotted in Fig. 4. Notice that the fast initial activation is due to the increased diffusion of the fast-activating layer. An extended analysis of convergence

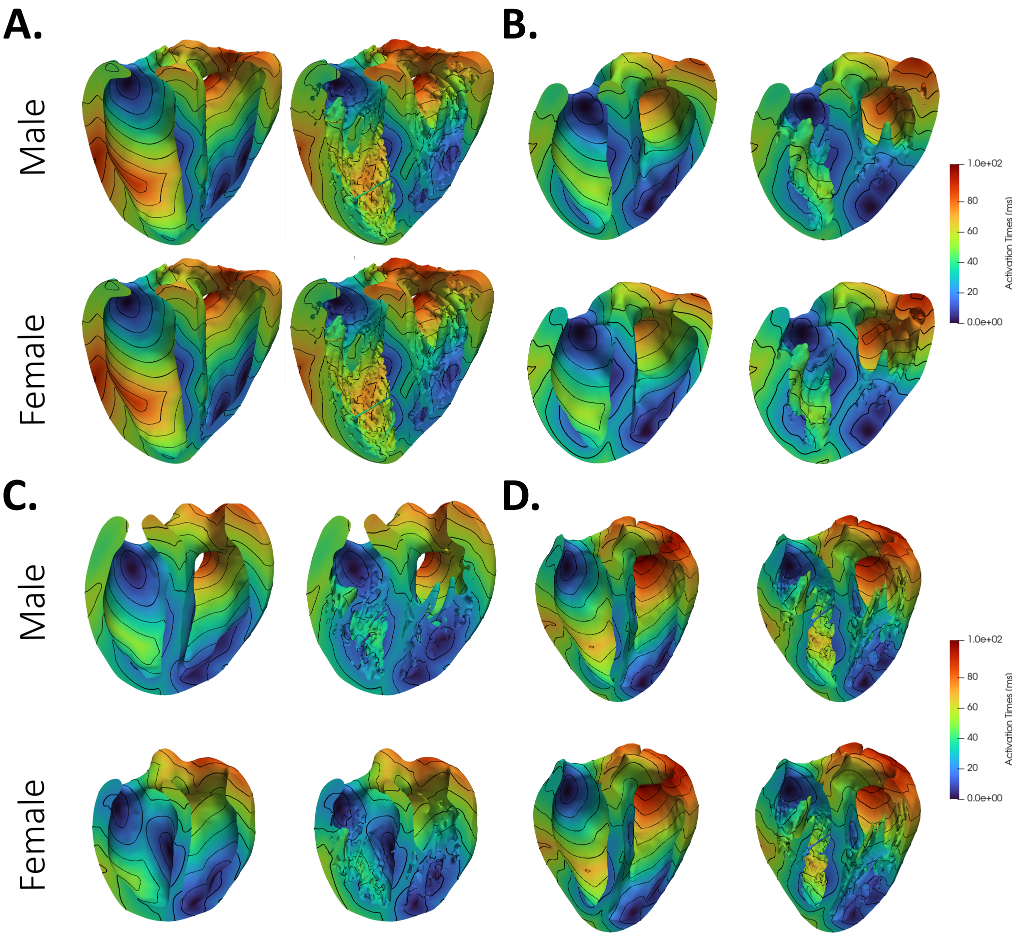

Fig 3. Isochrones of the activation propagation in all anatomies for male and female phenotypes.

given a variety of diffusion coefficients using Alya can be found in [8]. Based on these results, the verification of the convergence of the model was ensured.

Table 2. Numerical verification.

	No. Elements	Edge Length (cm)	Conduction Velocity (cm/s)
Initial mesh	20664	0.07	44.375
Subdivision 1 (div1)	165312	0.035	71.0
Subdivision 2 (div2)	1322496	0.0175	73.44

S1.6 Scar registration.

To register scar from patient MRI to detailed anatomy MRI, ANTS [9] was used. The registration was applied to the segmentation masks (0-background, 100-foreground) resampled at 0.1mm isotropic voxel size using bicubic interpolation. The transformations were rigid, affine, followed by the deformable SyN [10] with 3 stages. The default parameters of the ”antsRegistrationSyNQuick.sh” script were used, except the SyN parameters that were changed to $[0.1, 8, 0]$ to obtain a visually acceptable alignment of

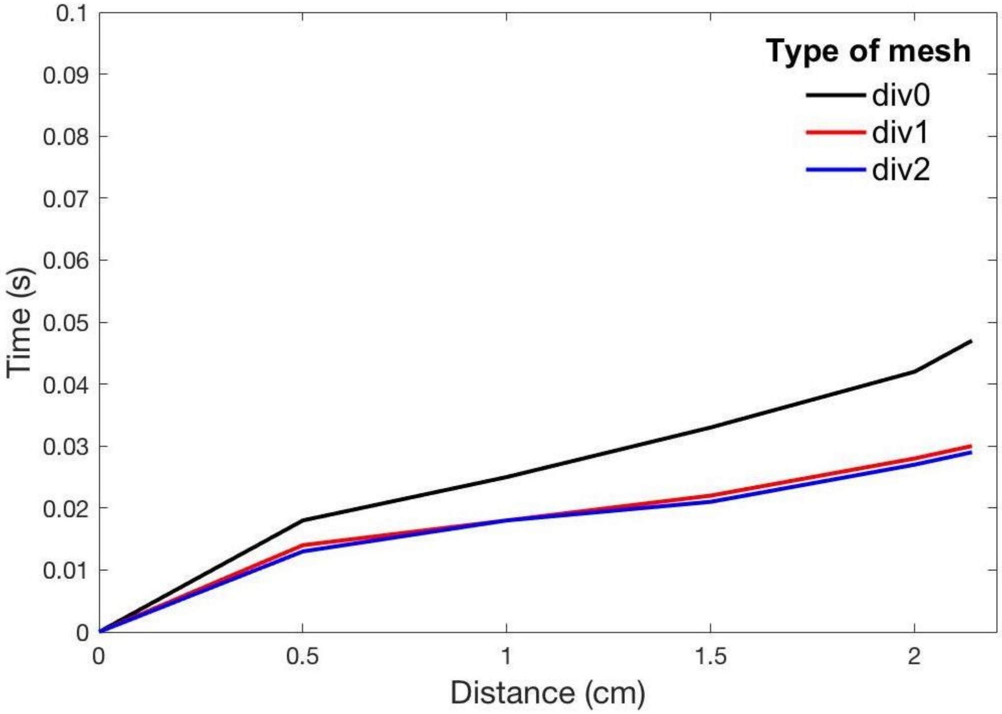

Fig 4. Electrophysiological model verification. Activation time is plotted against the distance to the origin on the diagonal direction in a slab. The curves corresponding to div1 and div 2 only show a 3% difference.

the dense myocardium. The calculated transform was used to warp the scar information. Fig. 5 shows the registered masks (MRI with scar to detailed MRI). The resulting registered scars on both the detailed and smooth anatomies can be observed in Fig. 6. It is clear that differences between the scars registered in the two anatomies are negliglible.

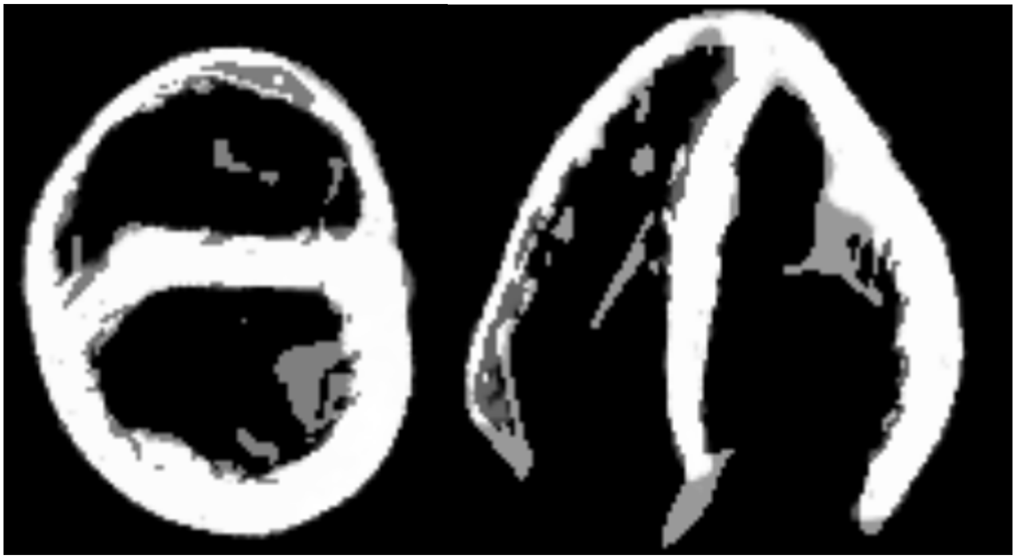

Fig 5. Overlaid registered masks (MRI with scar to detailed MRI).

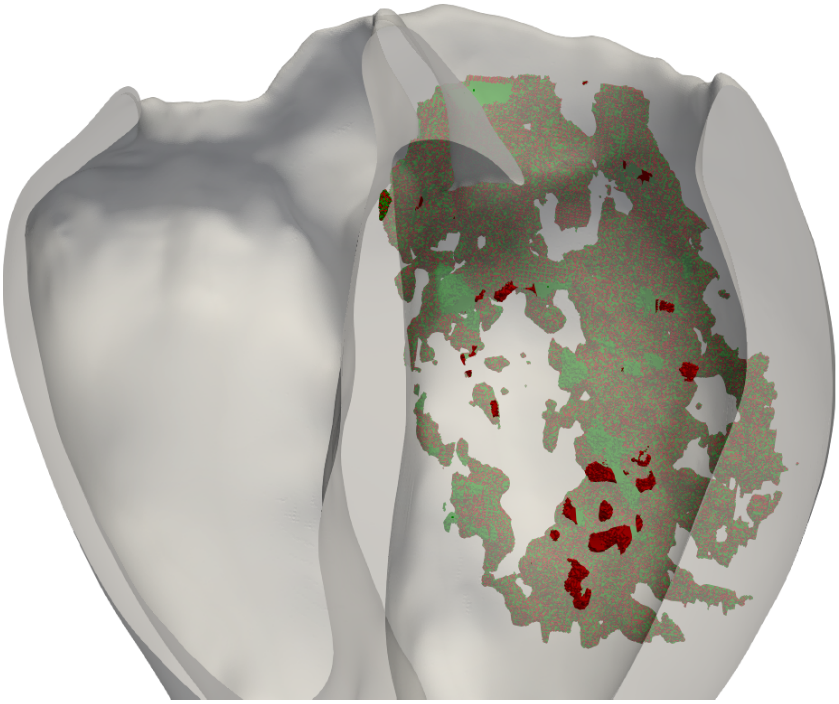

Fig 6. Overlaid scar registered on the smooth and detailed anatomies. Green corresponds to the scar in the smooth anatomy, red to the detailed anatomy.

S3 Video. Ventricular tachycardia media.

All videos are publically available at the following DOI for Figshare data: `10.6084/m9.figshare.11908029`
Video S1: Ventricular Tachycardia generated on a female phenotype simulation after RV Apex S1-S4 programmed stimulation protocol. Anatomical data was obtained from high resolution MRI ex-vivo human hearts.
Video S2: Ventricular Tachycardia generated on a female phenotype simulation on a smoothed geometry after RV Apex S1-S4 programmed stimulation protocol.
Video S3: Ventricular Tachycardia generated on a male phenotype simulation on a detailed geometry after LV Apex S1-S4 programmed stimulation protocol.
Video S4: Ventricular Tachycardia generated on a male phenotype simulation on a smoothed geometry after RV Apex S1-S4 programmed stimulation protocol.
Video S5: Ventricular Tachycardia generated on a male phenotype simulation on a smoothed geometry after RVOT Apex S1-S4 programmed stimulation protocol.

References

1. Schindelin J, Arganda-Carreras I, Frise E, Kaynig, et al. Fiji: an open-source platform for biological-image analysis. Nat Meth. 2012;9(7):676–682.
2. Qi C. Maximum Entropy for Image Segmentation based on an Adaptive Particle Swarm Optimization. Appl Math Inform Sci. 2014;8(6):3129–3135.
3. CIBC. Seg3D: Volumetric Image Segmentation and Visualization. Scientific Computing and Imaging Institute. 2016;.

4. Attene M, Falcidieno B. ReMESH: An Interactive Environment to Edit and Repair Triangle Meshes. IEEE International Conference on Shape Modeling and Applications. 2006; p. 41–41.

5. Santiago A, Aguado-Sierra J, Zavala-Aké M, Doste-Beltran R, Gómez S, Arís R, et al. Fully coupled fluid-electro-mechanical model of the human heart for supercomputers. International Journal for Numerical Methods in Biomedical Engineering. 2018;34(12):e3140. doi:10.1002/cnm.3140.

6. Niederer SA, Kerfoot E, Benson AP, Bernabeu MO, Bernus O, Cherry EM, et al. Verification of cardiac tissue electrophysiology simulators using an N-version benchmark. Philos Trans A Math Phys Eng Sci. 2011;13(369):4331–51.

7. Sacco F. Quantification of the influence of detailed endocardial structures on human cardiac haemodynamics and electrophysiology using HPC. Universitat Pompeu Fabra; 2019. Available from: `http://hdl.handle.net/10803/667670`.

8. López-Yunta M. Multimodal ventricular tachycardia analysis : towards the accurate parametrization of predictive HPC electrophysiological computational models. UPC, Facultat de Matemàtiques i Estadística; 2018. Available from: `http://hdl.handle.net/2117/124702`.

9. Avants B, Tustison N, Song G. Advanced Normalization Tools: V1.0. The Insight Journal. 2009;doi:10.54294/uvnhin.

10. Avants BB, Epstein CL, Grossman M, Gee JC. Symmetric diffeomorphic image registration with cross-correlation: evaluating automated labeling of elderly and neurodegenerative brain. Medical Image Analysis. 2008;12(1):26–41.
